# Association of mutation signature effectuating processes with mutation hotspots in driver genes and non-coding regions

**DOI:** 10.1038/s41467-021-27792-6

**Published:** 2022-01-10

**Authors:** John K. L. Wong, Christian Aichmüller, Markus Schulze, Mario Hlevnjak, Shaymaa Elgaafary, Peter Lichter, Marc Zapatka

**Affiliations:** 1 Division of Molecular Genetics and German Cancer Consortium (DKTK), German Cancer Research Center (DKFZ), Heidelberg, Germany; 2 Computational Oncology Group, Molecular Precision Oncology Program, National Center for Tumor Diseases (NCT) and DKFZ, Heidelberg, Germany; 3 Gynecologic Oncology, National Center for Tumor Diseases (NCT) and University of Heidelberg, Heidelberg, Germany; 4 Molecular Precision Oncology Program at the National Center for Tumor Diseases (NCT) and DKFZ, Heidelberg, Germany

**Keywords:** Oncogenes, Cancer genomics, Software

## Abstract

Cancer driving mutations are difficult to identify especially in the non-coding part of the genome. Here, we present sigDriver, an algorithm dedicated to call driver mutations. Using 3813 whole-genome sequenced tumors from *International Cancer Genome Consortium*, *The Cancer Genome Atlas Program*, and a childhood pan-cancer cohort, we employ mutational signatures based on single-base substitution in the context of tri- and penta-nucleotide motifs for hotspot discovery. Knowledge-based annotations on mutational hotspots reveal enrichment in coding regions and regulatory elements for 6 mutational signatures, including APOBEC and somatic hypermutation signatures. APOBEC activity is associated with 32 hotspots of which 11 are known and 11 are putative regulatory drivers. Somatic single nucleotide variants clusters detected at hypermutation-associated hotspots are distinct from translocation or gene amplifications. Patients carrying APOBEC induced *PIK3CA* driver mutations show lower occurrence of signature SBS39. In summary, sigDriver uncovers mutational processes associated with known and putative tumor drivers and hotspots particularly in the non-coding regions of the genome.

## Abstract (web summary)

In cancer, associations between mutational signatures and driver mutations have been proposed but not fully explored. Here, the authors develop sigDriver to find associations between mutational signatures and mutation hotspots in order to predict coding and non-coding driver mutations in pan-cancer genomics data.

## Introduction

International Pan-Cancer genome consortia such as the *International Cancer Genome Consortium* (ICGC)^1^, *The Cancer Genome Atlas* (TCGA)^2^, and the Pan-cancer analysis of childhood cancers^3^ open up the opportunity for large scale data-mining in the non-coding region of the human genome, as demonstrated by recent efforts within *The Pan-cancer Analysis of Whole Genomes* (PCAWG) initiative^4^. As cancer driving mutations are under positive selection during tumorigenesis, signals of positive selection are exploited for the discovery of drivers. The analysis of pan-cancer datasets revealed non-coding cancer drivers of lower frequency^5^ and advanced our understanding of mutational signatures^6^. The large number of passenger mutations found especially in whole-genome sequencing data of cancer samples complicate the effort to identify drivers^7^. So far sophisticated algorithms were designed to model the background mutation rate (BMR), an indicator for assessment of positive selection and for discriminating drivers from passengers^8–11^. The diversity of non-coding elements further complicates the estimation of BMR. Specialized models on distinct non-coding elements were designed recently to overcome the problem^10^. Meanwhile, some mutational signatures showed dependencies on driver mutations^12^, a phenomenon rarely considered by methods for driver discovery.

Mutational processes leave large footprints on cancer genomes, which are assessed on the basis of the frequency of nucleotide context of substitutions. Mutational signatures are defined by the proportion of mutations falling into mutation classes predefined by their nucleotide context^13^. They have been characterized in human case-control studies^14^ and were validated in cell line models^15^. The number of somatic mutations found in whole genomes empowered the detection of multiple mutational processes active in cancerogenesis^16^. Mutational signatures provide links to such processes manifested in the tumor genome during tumor development or treatment^17^.

Trinucleotide context information was used to define single-base substitution (SBS) mutational signatures^18^. The increasing number of reported trinucleotide mutational signatures poses challenges to consistent signature assignment^12^ as different methods provide divergent results^19^. Also more context information like DNA strand information or penta-nucleotide context might allow to differentiate further signatures. Our here presented driver identification approach is incorporating additional signatures using only N’s in NxSxN penta-nucleotide context information regardless of the nucleotide ‘x’s, where S is the position of the mutated base, x are the omitted neighboring bases, and N are two bases apart from the mutated single nucleotide used together with the S position in the analysis. This approach reduced the penta-nucleotide context information complexity from 1536 classes (6x4x4x4x4) to 96 classes (6x4x4) while enlarging the footprint of the motif and thereby covering additional mutational processes with a wider motif dependency. We here show that including those additional mutational signatures leads to identification of further putative tumor drivers.

Recent driver discovery algorithms consider the local nucleotide context to improve performance^20^. Some have taken the approach of manually curating candidate drivers tied to known mutational processes^5^. Alternatively, features related to hairpin structures were suggested for APOBEC mutagenesis to distinguish between drivers and passenger mutations^21^.

**Fig. 1:**
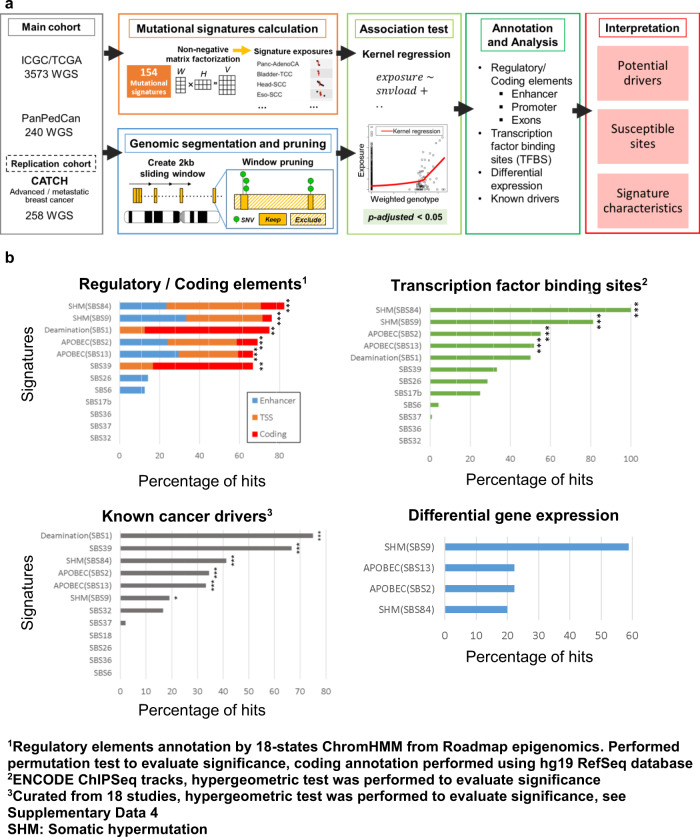
Association analysis reveals hotspots associated with mutational signatures. **a** A flow chart on driver discovery using hotspot-signature association on the basis of 3813 whole-genome sequenced tumors and replication using 258 whole-genome sequenced breast tumors. **b** Associated hotspots per mutational signature were annotated by coding regions, regulatory elements (enhancers and transcription start sites, TSS), transcription factor binding sites, known cancer drivers and gene expression changes. Enrichment tests were performed on coding elements and regulatory elements together by permutation. Hotspots enrichment on transcription factor binding sites and known drivers were performed by two-sided hypergeometric tests. Signature-set wise correction of *p* values was performed by false discovery rate (Benjamini-Hochberg, Supplementary Data 4). The significance level indicated by *** and ** are *q* < 0.001 and *q* < 0.01 respectively. Source data are provided as a Source Data file.

Here we present a driver discovery method associating sSNV (somatic single nucleotide variant) hotspots with mutational signatures. The method aims at identification of candidates by driver dependencies of mutational signatures and enriched targets of mutational processes (see Fig. 1a for an overview). Using published and additionally derived sSNV signatures, we detected associated hotspots down to the single-base resolution. By applying streamlined knowledge-based and annotation-based filters on mutation hotspots, putative driver mutations were distinguished from passenger mutations. The Roadmap Epigenomics Consortium^22^ provided essential references for the annotation of non-coding regulatory elements. So far, several attempts were made to link driver mutations to specific mutational signatures^23,24^. The here presented general approach is applicable to all SNV mutational signature sets and could reduce the need of manual curation when considering putative drivers involved in mutational processes^5^. We use enrichment of regulatory or coding elements per mutational signature and the recurrence per hotspot as an indicator for positive selection, in contrast to commonly used BMR models adjusted for local mutational processes. We define a mutational process to be driver associated if we find an event significantly enriched in known drivers and in coding/regulatory regions with more than three events overlapping with COSMIC cancer census genes. We demonstrate that specific mutational processes are linked to higher rates of driver mutations. In addition, we reveal potential non-coding drivers, and suggest possible causes of some hitherto uncharacterized signatures. In this work, we present sigDriver a tool developed to identify mutational processes associated with known and putative tumor drivers and hotspots particularly in the non-coding regions of the genome to predict coding and non-coding driver mutations.

## Results

A total of 3813 human tumors and matching germlines from three whole-genome sequencing cohorts were used as discovery data set, including 2,772 from ICGC^1^, 801 from TCGA^2^, and 240 from a pan-pediatric cancers cohort^3^, of which 2,702 are part of PCAWG^6^. These data set yielded 52,382,573 somatic SNVs (sSNVs) and 154 substitutional mutation signatures from three sets of signature definitions (50 COSMIC SBS trinucleotide signatures, 61 penta-nucleotide signatures, 43 NxSxN-extended signatures). For driver discovery, sigDriver scans the target genome by half-overlapping windows of 2 kilobases (kb). Within each 2 kb region, signature-associated hotspots were resolved down to single-base resolution. Each hotspot was regressed to search for signature-associated regions. Mutational load was controlled to avoid spurious association. Differential expression analysis and annotation of epigenetic and genomic elements were applied to each hotspot in order to evaluate their cancer driver potential. Interestingly, distinct mutational processes contributed to substitutions preferentially in coding regions or regulatory DNA elements (Supplementary Data 2), showed enrichment of events on known drivers of which more than three events overlap with Cancer Gene Census genes^25^. This type of mutational process is considered as driver-associated mutational process whereas the corresponding mutational signature is referred as driver-associated mutational signature. In addition, by comparing all signature-associated hotspots in regulatory regions with those that are not, we found a significant higher number of differential expression features in regulatory regions (*p* < 0.020, chi-square test, Supplementary Data 3). A signature-associated hotspot was classified as putative driver if it was significantly associated with a driver-associated mutational process and, in addition, meet at least one of the following criteria: (1) is located in a regulatory element (enhancers, promoters), (2) is located in a coding region, (3) affects gene expression (including transcript distribution), or (4) is highly recurrent (point mutations present in at least 10 cases per mutational signature tested) (Fig. 1b).

### Overview of hotspot mutations associated with various mutational processes

**Fig. 2:**
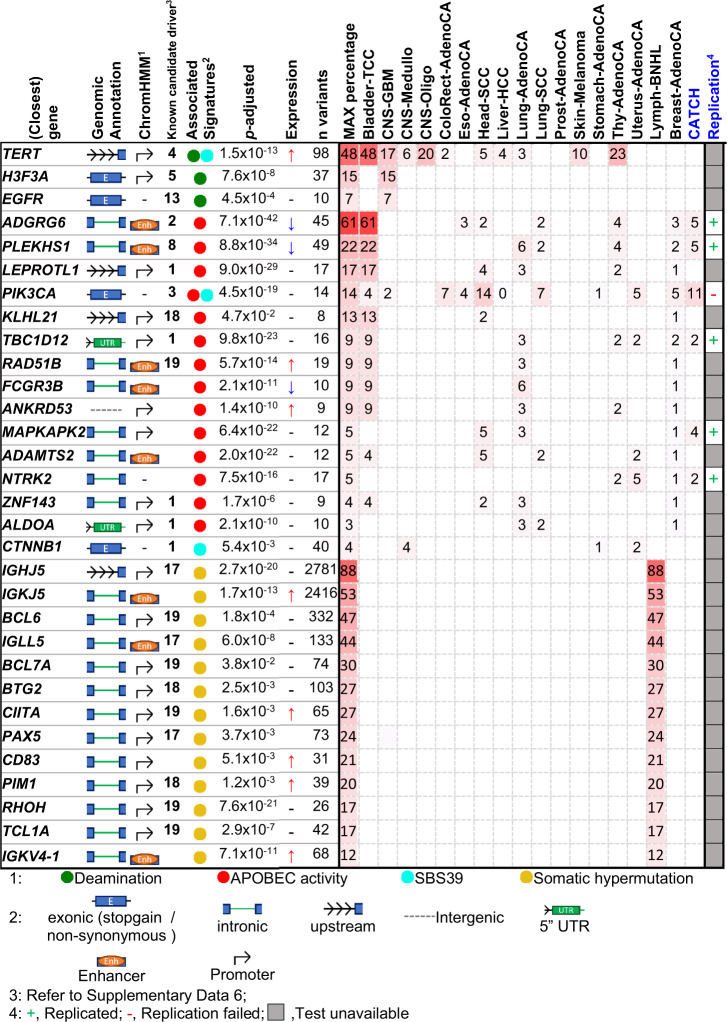
A summary of putative driver hotspots from COSMIC SBS mutational signatures V3. Hotspots from six driver-associated mutational signatures were included, signatures are classified into 4 categories: Deamination (SBS1), APOBEC (SBS2/SBS13), somatic hypermutation (SBS9, SBS84), and signature SBS39. Hotspots were annotated by the nearest gene, by their expression impact, by their epigenetic impact from ChromHMM, and by their status as known drivers. The *p* values indicate the significance level calculated by sigDriver of the corresponding hotspot after multiple testing correction. Occurrence across entities were summarized in percentages on the heatmap section of the table. The replication cohort designated CATCH comprises metastatic breast cancer samples (blue); the replication status is provided in the respective column. Source data are provided as a Source Data file.

We identified six signatures that are associated with driver mutations based on hotspots enrichment tests (Supplementary Data 4), including *APOBEC*-driven mutagenesis (SBS2, SBS13), spontaneous deamination (SBS1), somatic hypermutation (SBS9, SBS84) and an uncharacterized signature (SBS39). For details on other mutational signatures, refer to Supplementary Note 4. Of 84 hotspots originating from these mutational processes, 70 are putative drivers and 40 are known drivers (Supplementary Data 5, literature references in Supplementary Data 6). Top putative drivers per mutational signature were summarized in Fig. 2. Our tool identified only functional hotspots in the coding region, consisting of non-synonymous or nonsense sSNVs. Coding drivers were delineated by *H3F3A* and *PTEN* hotspots from spontaneous deamination (Fig. S1) and by two distinct hotspots on *PIK3CA* resulting from APOBEC activity and signature SBS39, respectively. Additionally, all 19 of our unique coding hotspots (6 from NxSxN-extended signatures) were also reported by PCAWG investigations of coding drivers^5^ (Supplementary Data 12, 13), demonstrating the underlying specificity of our driver discovery method. Our approach is, however, universal to identify coding and non-coding driver candidates over the entire genome.

While all coding hotspots from driver-associated mutational processes (SBS1, SBS2/SBS13, SBS39, SBS9/SBS84) are known, non-coding hotspots are largely undescribed (23 of 49, Supplementary Data 5). Identified *TERT* promoter mutations are known and associated with the spontaneous deamination signature (SBS1, adj*. p* value *<* 4.5 × 10^−8^, Supplementary Fig. 1). APOBEC mutagenesis (SBS2, SBS13), somatic hypermutation (SBS9, SBS84) and spontaneous deamination (SBS1) play an active role in driver mutagenesis, particularly in the non-coding region. APOBEC activity is accountable for multiple known non-coding driver mutations affecting *PLEKHS1, TBC1D12, LEPROTL1*, and *ADGRG6*^26–28^, along with so far undescribed drivers identified by sigDriver nearby: *MAPKAPK2, RAD51B, ADAMTS2*, and *NTRK2*. Somatic hypermutation signatures (SBS9, SBS84) are associated with a small subset of activation-induced cytidine deaminase (AID) targeted regulatory hotspots.

### APOBEC-associated hotspots

**Fig. 3:**
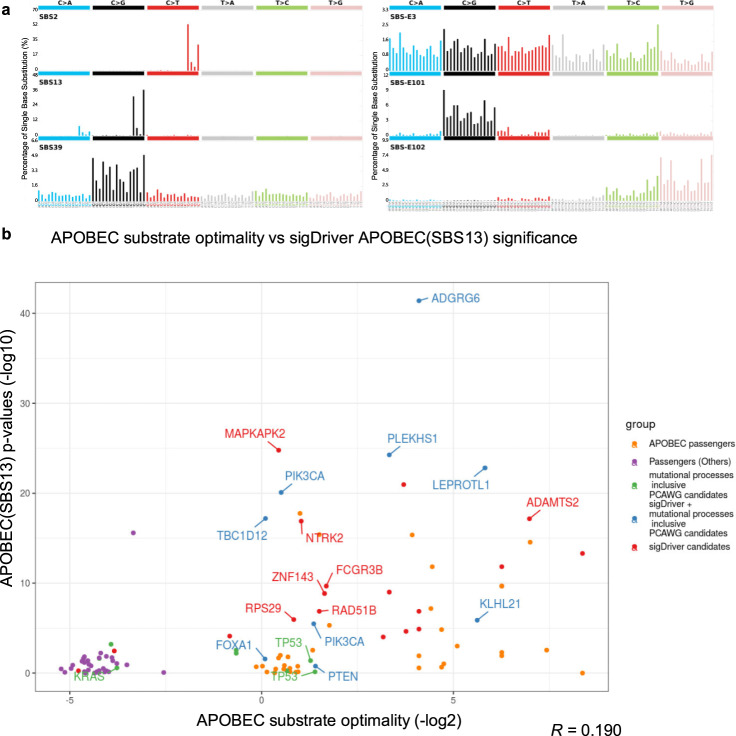
Selected mutation spectra of COSMIC SBS V3 and NxSxN signatures and analysis of  APOBEC substrate optimality in relation to sigDriver APOBEC(SBS13) significance. **a** The mutation spectrum of signatures from COSMIC SBS V3 signatures and NxSxN-extended signatures. The left panel is showing the mutation spectrum of the APOBEC signatures (SBS2/SBS13) and signature SBS39 under the COSMIC SBS V3 signature definition. The right panel is showing the mutation spectrum of the defective homologous recombination signature SBS-E3 and signatures SBS-E101, SBS-E102 in the NxSxN-extended signature definition. **b** APOBEC substrate optimality analysis on recurrent hotpots tested by sigDriver. Hotspots were extracted from APOBEC positive tumors (more than 5% activity) for APOBEC substrate optimality analysis by ApoHP^30^. Potential drivers reported by PCAWG, sigDriver and the overlap of the two were annotated per hotspot. Spearman’s correlation between the APOBEC substrate optimality and the sigDriver significance was used to assess the relationship between the two. Source data are provided as a Source Data file.

APOBEC induced signatures are associated with the highest numbers of putative driver mutations. In total, 34 associated hotspots were linked to APOBEC signatures (SBS2, SBS13, SBS-E2/E13, Supplementary Data 7, Supplementary Fig. 2). A literature review showed that 11 of the 34 hotspots (32%) are known drivers and are amongst the top hotspots linked to APOBEC signatures (Supplementary Data 7). Five of the seven identified APOBEC-associated hotspots were replicated in an independent cohort of metastatic breast cancer (Supplementary Data 8). The graphical illustration of the mutation spectrum of APOBEC signatures (SBS2/SBS13) is presented in Fig. 3a. All point mutations associated with APOBEC signatures showed the characteristic C > G, C > T and C > A substitutions confirming the link with this enzyme (Supplementary Fig. 2). Analyzing the DNA replication strand asymmetry at these sites, we annotated the leading and lagging strands for 6 out of 34 APOBEC hotspots with 4 hotspots on the lagging strand (Supplementary Data 7). The finding is in line with the observation that APOBEC mutagenesis primarily occurs on the lagging strand template during DNA replication^29^. Substrate optimality prediction^21^ revealed that APOBEC hotspots are at least weak substrates of APOBEC enzymes (optimality > 0), while the hotspot *p* values are only weakly correlated with substrate optimality (*r* = 0.190, Spearman’s Correlation, Fig. 3b). Some proposed driver hotspots nearby *NTRK2* and *MAPKAPK2* are low in APOBEC optimality (<1.1, Supplementary Data 7). Major APOBEC-associated hotspots are described in Supplementary Fig. 2. This suggests that sigDriver can distinguish APOBEC-associated drivers from other drivers without being confounded by the optimal substrates of APOBEC.

APOBEC-associated hotspots are enriched for regulatory elements, coding elements, transcription factors binding sites and known tumor drivers (Fig. 1b). Enrichment for regulatory elements was most pronounced (20 of 34, Supplementary Data 7, coding: *p* < 0.002, regulatory: *p* < 1 × 10^−6^, permutation test). Using ChromHMM 18-state annotations from 98 epigenomes^22^, we found significant enrichment of enhancer or promoter elements in hotspots associated with APOBEC signatures (SBS2 and SBS13, adj*. p* value < 1.1 × 10^−4^). Exploration in the non-coding region for APOBEC hotspots sigDriver can identify candidate drivers like *ADGRG6* among the genes identified as likely passengers^30^.

APOBEC initiated mutagenesis is common across multiple tumor entities^6^. However, entity-specific positive selection causes differences in prevalence of APOBEC induced driver mutations (Supplementary Data 9). We observed a high frequency of bladder-transitional cell carcinomas (Bladder-TCC) with mutations at APOBEC hotspots (20 of 34). Hotspots in *NTRK2* or *MAPKAPK2* are not present in bladder carcinoma but in other entities (Head-SCC, Lung-AdenoCA and Uterus-AdenoCA). *MAPKAPK2* hotspots were shared across three entities, namely breast carcinoma (7 of 644 tumors), head and neck squamous cell carcinoma (3 of 56 tumors) and cervical squamous cell carcinoma (1 of 18 tumors). Most APOBEC hotspots are found in breast carcinoma (33 of 34 hotspots) where APOBEC activity is common.

The APOBEC hotspot on *MAPKAPK2* is characterized by C > T or C > G substitutions at chr1:206859376 overlapping with the promoter region of *MAPKAPK2* (Supplementary Fig. 3). The variant is also present at low frequency in the general population (gnomAD allele frequency = 0.00152, rs180968364). It is supported by 12 sSNVs across 4 tumor entities (Breast-AdenoCA, Lung-AdenoCA, Head-SCC, and Cervix-SCC). Interestingly, this mutation is not identified in bladder carcinoma, even though this entity is associated with high APOBEC activity. According to the algorithm DeepBind, three transcription factors are predicted to have reduced binding probability in that region: CHD2, ZBTB33, and BRCA1 (Supplementary Data 10). The respective binding sites are active as indicated by ChIP-seq experiments^31^ and the corresponding transcription factors are also expressed in bladder carcinomas^32^. The APOBEC activity predominantly substituted C to T within the core CGCG repeat of the ZBTB33 (Kaiso) binding site. The mutation could potentially disrupt the co-repressor activity of Kaiso for activation of *MAPKAPK2*. The weak APOBEC substrate optimality of the hotspot (1.36) further supports it as driver candidate as hotspots with a score below 4 are known or likely drivers^30^. This hotspot was replicated in an independent cohort of metastatic breast cancer (9 of 258 tumors, 3.5%, Supplementary Data 8), where the occurrence is higher than that of primary breast cancers (7 of 640, 1.1%, Supplementary Data 9). Although the hotspot mutation does not significantly affect *MAPKAPK2* expression, it is associated with the downregulation of *DYRK3* (PCAWG, *q* < 0.036, CATCH *p* < 0.003, Supplementary Fig. 4). In line with this finding is the GeneHancer database^33^ that describes this regulatory region on *MAPKAPK2* regulating the nearby *DYRK3* gene, a gene within the topologically associating domain (TAD) boundaries of the hotspot.

### Hotspots associated with spontaneous deamination signature

The spontaneous deamination signature (SBS1) is based on a mutational process correlated with age^6^. Hotspots were investigated for their relationship with age to understand whether the putative driver mutations are acquired over time. The majority of hotspots associated with the spontaneous deamination signature (SBS1) are coding with functional impact (Fig. 1b), affecting known drivers such as *PTEN, EGFR*, and *H3F3A* (Supplementary Data 5 and Supplementary Note 3). However, no link with age can be found over the hotspots associated with the spontaneous deamination signature (Supplementary Data 11).

Apart from the six driver-associated signatures, hotspots from other mutational signatures showed less overlap with known regulatory elements. These COSMIC SBS signature hotspots only accounted for 17% (14 of 82) of all hotspots identified on coding DNA or regulatory elements (Supplementary Data 12). They are referred to as susceptible targets of the underlying mutational process.

### The interplay between *PIK3CA* activating mutations and mutational processes

**Fig. 4:**
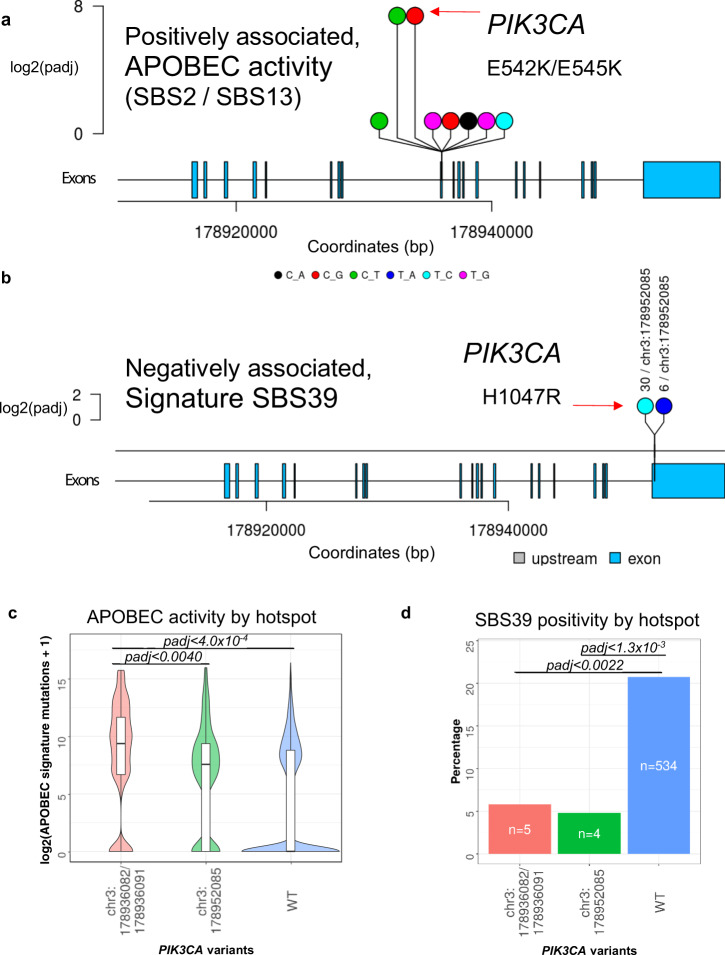
Relationship between *PIK3CA* hotspot mutations and mutational signature exposures. **a** *PIK3CA* hotspot on exon 12 positively associated with APOBEC signatures (SBS2/SBS13). **b** *PIK3CA* hotspot on exon 1 negatively associated with signature SBS39. **c** Violin-plot comparing APOBEC signature exposures (SBS2+SBS13) on *PIK3CA* hotspots, significance between the wild-type (WT) group and the mutated group was tested by a two-sided Wilcoxon rank-sum test (*p* < 2.0 × 10^−4^ and *p* < 0.0020). The box indicates the 25th and 75th percentiles with the median highlighted by a black line, whiskers extend to 1.5 times the interquartile range from the 25th and 75th percentiles, and polygons represent density estimates of data. **d** Bar-plot comparing the binary status of signature SBS39 exposures in tumors carrying different hotspot mutations. Tumors with more than 5% normalized signature exposures were considered positive, significance between the wild-type (WT) group and the mutated groups were evaluated by a two-sided chi-square test and corrected for multiple testing according to Bonferroni (*p*_adj_<1.3 × 10^−3^ and 0.0022). Source data are provided in Supplementary Data 4.

Two hotspot mutations were detected in the oncogene *PIK3CA* and are linked to two independent mutational signatures. While the hotspot p.E542K/p.E545K is associated with APOBEC signatures (SBS13, adj*. p* value *<* 1.0 × 10^−16^), the hotspot p.H1047R is negatively associated with signature SBS39 (adj*. p* value < 0.018, Supplementary Data 5) and with homologous recombination deficiencies (SBS-E3, adj*. p* value *<* 2.2 × 10^−5^). The p.E542K/E545K hotspot is known to be raised by APOBEC with the characteristic C > T substitution on the antisense strand^34^. We observed that tumors carrying p.E542K/E545K mutations have significantly higher fraction of APOBEC signature linked mutations than p.H1047R carriers (Fig. 4c). Meanwhile, tumors carrying these hotspots have a lower occurrence of signature SBS39 (p.H1047R, adj*. p* value < 1.3 × 10^−3^, Fig. 4d). Both hotspot mutations are activating driver mutations of the PIK3CA kinase affecting the helicase or the kinase domain^35^. By investigating the presence of APOBEC signatures (SBS2/13) and signature SBS39 in breast cancer subtypes defined by TCGA^36^, we found a significant enrichment of SBS39 exposures in the basal subtype compared with 3 other breast cancer subtypes defined by PAM50 (Her2, Luminal A, Luminal B; Supplementary Fig. 5). By our independent cohort of advanced breast cancers, we observed a weak negative correlation between APOBEC induced and SBS39 signatures (*r* = −0.27, spearman). An interplay between the mutational signatures (APOBEC and SBS39) was observed through hotspot mutations in *PIK3CA*.

### Additional hotspots discovered by NxSxN-extended and penta-nucleotide motifs

By extending the analysis to additional signature sets, additional hotspots were discovered. Some were exclusive to NxSxN-extended signatures (SBS-E) (Supplementary Data 13), as exemplified by known coding driver hotspots at *TP53* (SBS-E3), and two coding hotspots exclusively identified by its somatic hypermutation signature (SBS-E9) (Supplementary Data 26). A total of 59 hotspots were identified using NxSxN-extended signatures with 10 hotspots showing a maximum entity prevalence of more than 3% (Supplementary Data 14). Of the 115 unique hotspots discovered using NxSxN-extended signatures, 60 are also present among the identified COSMIC SBS hotspots (Supplementary Fig. 6). Additionally, NxSxN-extended signatures exclusively identified coding hotspots with functional impact on *SPOP* and *SMARCA4*. Signatures SBS-E102 and SBS-E108 provided the most complementary information as compared to COSMIC SBS signatures (Supplementary Data 13). Homologous recombination deficiency signatures SBS-E3 showed a higher number of putative driver hotspots than SBS3, but there is insufficient evidence to classify SBS-E3 as driver-associated. In conclusion, utilization of NxSxN-extended signatures resulted in additional hotspots including 11 known drivers missed from COSMIC SBS signatures analysis (Supplementary Data 13).

Although full penta-nucleotide signatures accounted for more context information, only 47 additional hotspots were added to the classical COSMIC SBS signatures (Supplementary Data 13). Of all the penta-nucleotide associated hotspots, *BRAF* and *FOXO1* from PSBS69 and PSBS71 are notable examples unaccounted for by COSMIC SBS signatures. The *BRAF* signal originates from V600E coding mutations in glioblastoma, a potential biomarker for drug response in glioblastoma^24^. Penta-nucleotide signatures can therefore extract information missed by using only COSMIC trinucleotide SBS signatures for analysis.

We here report hotspots discovered by NxSxN-extended signatures and by penta-nucleotide signatures (Supplementary Data 15). The overlapping hotspots between the two signature sets are 44% (46 of 105 Penta-nucleotide signature hotspots, Supplementary Fig. 6). Surrogate signatures showed consistency of the method despite differences in signature construction (Supplementary Fig. 7). The inclusion of additional signature sets can benefit driver discovery by two aspects: repeated measurements by surrogate signatures and extraction of missing information by so far disregarded signatures.

### Differential expression analysis comparing mutational status of hotspots

Using expression data from 1359 tumors of PCAWG, the impact of the hotspot mutations on gene expression was evaluated. Tests were performed across all entities and on each entity when sufficient number of tumors were available (*n* > = 3). Gene copy numbers were controlled to focus on the effect from hotspots.

**Fig. 5:**
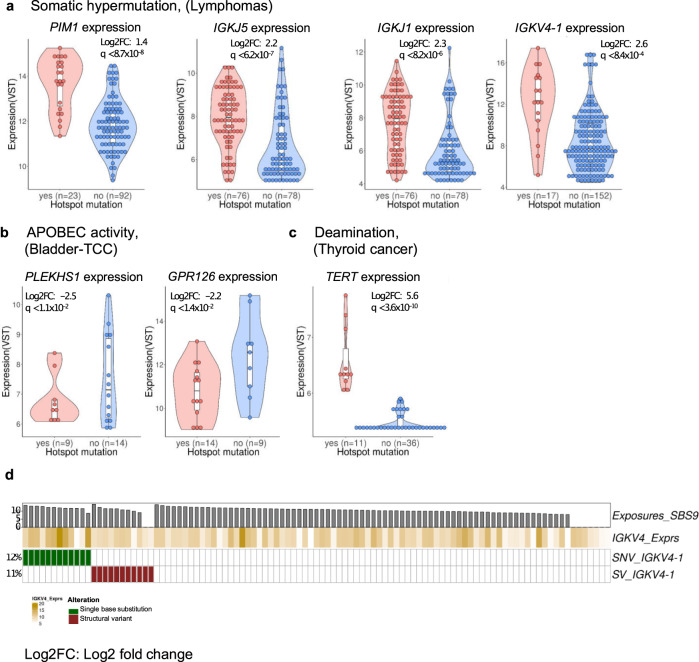
Gene expression changes related to putative driver hotspots. Significance and log_2_ fold changes for two-sided tests were provided by DESeq2 and presented values are VST transformed. *p* values were FDR corrected by the total number of expression tests performed on the COSMIC SBS signature set, indicated by the *q*-values. For details of the hotspots, refer to lollipop plots in the Supplementary Figs. 2, 3, and 7. In the violin plots, the white box indicates the 25th and 75th percentiles with the median highlighted by a black line, whiskers extend to 1.5 times the interquartile range from the 25th and 75th percentiles, and polygons represent density estimates of data and extend to extreme values. **a** Expression boxplots for *PIM1, IGKJ5, IGKJ1,* and *IGKV4-1* from lymphomas (Lymph-BNHL and Lymph-CLL) associated with somatic hypermutation (SBS84, SBS9), contrasting tumors with and without hotspot mutations. **b** Expression boxplots for *PLEKHS1* and *ADGRG6 (GPR126)* from bladder cancer (Bladder-TCC) associated with APOBEC activity (SBS2/SBS13). **c** Expression boxplots for *TERT* hotspot associated with spontaneous deamination (SBS1). **d** Oncoprint on mutation status of *IGKV4-1* in B-cell non-Hodgkin lymphoma (Lymph-BNHL), describing the relationship between structural variants (SVs) within 200 kb and single nucleotide variants (SNVs) on the hotspot. Source data are provided as a Source Data file and in Supplementary Data 16.

Twenty-five of 90 hotspots from COSMIC SBS signatures showed differential gene expression after false discovery rate correction (Supplementary Data 16). Likewise, in our study 20 of 65 hotspots showed differential transcript expression (Supplementary Data 17). Deregulations were observed on genes such as *TERT, PLEKHS1,* and *ADGRG6* (Fig. 5b, c) or *RAD51B* and *FCGR3B* (both of them have hotspots located on the last intron, overlapping with regions annotated as enhancer). By intersecting with ChromHMM annotations, we detected that 23 of 25 gene expression changes coincided with mutations in regulatory elements defined by chromatin states (enhancer or promoter, Supplementary Data 18, Supplementary Fig. 8) and supporting the role of these hotspots as regulatory drivers. Hotspots associated with expression impact and within regulatory elements provide evidence for their potential role promoting tumor development.

Hotspots associated with somatic hypermutation are characterized by sSNVs clusters in promoter regions. Hotspots on regulatory regions of *IGKV4-1, CD83, CIITA, PIM1,* and *IGKJ5* are associated with the upregulation of the respective gene (Fig. 5a). Clusters of mutations were caused by somatic hypermutation, in contrast to APOBEC signature-associated hotspots consisting of only 1 to 2 specific point mutations (Supplementary Fig. 9). Over 80% of the hotspots associated with hypermutation activity are affecting promoter or enhancer elements defined by ChromHMM annotations (Fig. 1b). This is consistent with previously observed enrichment of somatic hypermutation hotspots at promoter regions^37^. To investigate whether our observation is due to immune cell maturation, tumors from the entity chronic lymphocytic leukemia (CLL) were further sub-classified by their IGHV mutational status^38^. The differentially expressed genes are unchanged with or without sub-classification by IGHV mutation status. By inspecting structural variants (SVs) nearby the hotspots of *IGKV4-1*, we observed that hotspot sSNVs are mutually exclusive to nearby SVs (Fig. 5e, Supplementary Data 19). Hotspot sSNVs associated with somatic hypermutation could be an alternative or synergistic mechanism of putative driver gene upregulation in Non-Hodgkin lymphoma (Lymph-BNHL).

### Comparison with other methods of driver discovery

To investigate the overlap between PCAWG identified candidate drivers with our list of putative drivers, mutational process-inclusive list of PCAWG candidate drivers were taken into account. To this list we added all driver candidates likely linked to mutational processes that were removed in the final PCAWG driver candidate list. All 12 of the identified coding candidate drivers are present in the mutational processes-inclusive list of PCAWG candidate coding drivers (*n* = 183, Supplementary Data 5). Notably, the overlap of mutational processes-inclusive non-coding candidate drivers from PCAWG (*n* = 188) with the candidate drivers from sigDriver (*n* = 70) is 16 (Supplementary Data 5). It is however difficult to evaluate the exclusive drivers from PCAWG and from our study, as the two approaches differ by the scope of genomic regions (functional/regulatory elements versus the entire genome) and the relationship of driver mutations with mutational processes (all driver mutations versus drivers associated with mutational processes).

We compared sigDriver with DriverPower, a method combining mutational burden and mutation impact for driver discovery^39^. At the false discovery rate of 10%, DriverPower reported 113 hits across coding and regulatory elements. Thirty-five of 113 hits are shared between DriverPower and sigDriver (Supplementary Data 20), of which 9 overlapping candidates from sigDriver were counted more than once due to the aggregation of coding and non-coding elements altogether in one test window. Overall, 45 of 70 hits are exclusive to sigDriver, of which 43 are categorized as non-coding elements. Since sigDriver is expected to reveal only putative drivers strongly associated with a mutational process, it detects less driver candidates. However, sigDriver presents a substantial addition of driver candidates to the non-coding region of the genome.

## Discussion

We performed a genome-wide search for mutational hotspots and drivers associated with three sets of mutational signatures. The set of NxSxN-extended signatures not only supported the findings but in addition lead to the discovery of putative drivers. In this context, we identified four mutational processes linked to six mutational signatures associated with driver mutations. Signatures were associated with mutational hotspots consisting of sSNVs, where knowledge-based annotations (chromatin states, coding regions) were applied to find putative drivers. As signatures of mutational processes can be found across multiple tumor entities, hotspot discovery can be performed on a collection of signature-positive entities. Our method took an automatized search-then-annotate approach to characterize hints of positive and negative selection implicated by dependencies and enriched targets of mutational processes, an approach different from using BMR models. Hotspots associated with mutational signatures were identified by mutational burden, whereas functional and regulatory annotations were applied to hotspots to evaluate driver potential.

Our approach attempts to distinguish driver from passenger mutations related to mutational processes. It revealed a number of potential non-coding drivers and suggested their link with one or more mutational signatures. The majority of those driver hotspots are cis-regulatory elements, located in regulatory elements of the target genes and yielding in gene expression alterations. Some hotspots are highly recurrent but unexplained by regulatory annotations, demonstrated by hotspots nearby *NTRK2* and *SLC14A2*. A subset of APOBEC activity linked hotspots were replicated in a smaller (n = 258) independent cohort of advanced metastatic breast cancer, assuring the reproducibility of the method. Additional non-coding drivers were identified^5^. SigDriver revealed known drivers in the coding sequence compartment without taking advantage of coding impact annotations. The characteristic annotation-free hotspot identification step enabled driver discovery in coding as well as under-investigated non-coding regions.

Six mutational signatures showed enrichment of hotspots with known drivers, regulatory and coding elements (SBS1, SBS2, SBS13, SBS39, SBS84, SBS9). APOBEC and somatic hypermutation hotspots are highly enriched for mutations in enhancer or promoter regions and are more likely to disturb transcription factor binding motifs. Deamination is instead more often found in coding regions. Physiological mutation processes, such as local hypermutations observed during immune cell maturation, could further increase hotspot at coding or regulatory elements.

To account for mutational processes that are known to target specific motifs or regulatory elements (SHM), we further performed APOBEC optimality analysis and IGHV mutational status analysis of our driver hotspots. This showed that sigDriver does not preferentially identify the favorite substrates of the mutational processes as drivers. A large proportion of hotspots from signature SBS84 (SHM) are overlapping with the COSMIC Cancer Gene Census (6 of 17)^25^. Putative drivers of chronic lymphocytic leukemia showed an increased gene expression regardless of IGHV mutational status. Moreover, the exclusivity of sSNVs and sSVs in *IGKV4-1* hint towards a positive selection during tumor evolution. These observations provide evidence for sigDriver-based hotspots in non-coding regions as putative drivers.

Complex interplay was observed between mutational processes and driver mutations. As an example, we observed patients carrying APOBEC-associated *PIK3CA* driver mutation (H1047R) are less likely to show SBS39 signature. Applying sigDriver, we have detected both positive and negative association between hotspots and mutational signatures, a potential determinant for the path of tumor evolution.

Some identified non-coding drivers apparently disrupt gene expression on the gene- or on the transcript-level. Non-coding driver mutations of *RAD51B* and *FCGR3B* are associated with disruption of isoform regulation. Promoter mutations of *ANKRD53* or SHM targets are associated with upregulation of gene expression, similar to the *TERT* gain-of-function promoter mutations. Likewise, hotspot sSNVs within an enhancer of *IGKV4-1* suggest a gene up-regulation mechanism. Some expression changes are detectable only in a subset of tumor entities, as demonstrated by the *PLEKHS1* hotspot in bladder cancer. It is foreseeable that by increasing cohort sizes of transcriptome sequencing efforts, the effects of these potential non-coding drivers on transcription regulation will be further uncovered.

Hotspots associated with mutational signatures provide a clue to the underlying mechanism for some uncharacterized signatures. Signatures SBS32/SBS37 are enriched for hotspots on LINE elements. By motif analysis on SBS17 hotspots, we found recurrent substitution within the motif AAAAC[T > G]TA, describing a characteristic feature of this otherwise poorly characterized signature.

Studying multiple mutation signature sets clearly improves the interpretation of associated hotspots and leads to additional driver discoveries. The penta-nucleotide information leads to increased complexity (6x4x4x4x4 = 1536 combinations) and gain of information^27^. Nevertheless, signatures reconstructed from NxSxN penta-nucleotides have the same complexity as trinucleotide signatures (6x4x4 = 96 classes), ensuring compatibility with existing signature analysis frameworks. Some NxSxN-extended signatures are surrogates of COSMIC SBS signatures, such as APOBEC and UV-light signatures. We here show that other - uncorrelated - NxSxN-extended signatures are a source for identification of additional mutation hotspots.

Our cancer whole-genome association studies using mutational signatures has led to candidate driver discoveries. This has been achieved despite the fact that mutational process associated drivers are entangled with passenger mutations. We anticipate that this approach will be further expanded by implementing further levels such as indel or methylome signatures. The forthcoming international efforts on large scale whole-genome sequencing will provide additional power for more discoveries by providing even larger sample collections.

## Methods

### Discovery cohorts

Somatic single nucleotide variants (sSNVs) information was retrieved from the ICGC data portal (dcc.icgc.org) and the R2 database (pedpancan.com), which were used to construct the driver analysis dataset. Summarized penta-nucleotide context information was retrieved from synapse database (https://www.synapse.org/#!Synapse:syn11801889) for NxSxN-extended signatures training. The driver analysis dataset is a subset of the signatures training dataset consist of 3813 tumors, where the full signature training set has 5070 tumors (Supplementary Data 1).

#### The NxSxN-extended signature training dataset

The NxSxN-extended signature training dataset is a combined set including the entire whole-genome sequencing cohort included in the PCAWG mutational signatures study (https://www.synapse.org/#!Synapse:syn11726601/files/), ICGC brain tumors and tumors in the pan-pediatric cancer cohort, summing up to 5070 tumors. The NxSxN penta-nucleotide information was extracted from the full 1536 classes of penta-nucleotides information provided in the synapse database and reduced into 96 classes of NxSxN penta-nucleotide context. For tumors not included in the PCAWG analysis, the 96 NxSxN penta-nucleotide classes were prepared by the preparation script provided by the PCAWG mutational signatures project, also by reducing the full 1536 classes of penta-nucleotides information to 96 NxSxN classes. See Supplementary Notes 1 and 2 for details.

#### The driver analysis dataset

The analysis dataset is a subset of the signature discovery dataset (*n* = 3813). For samples in this set, the genomic positions and the substitution type of somatic SNVs are available. Tumors from the ICGC data portal and in-house tumors passing quality control were included. The NxSxN penta-nucleotide information (96 classes) were extracted for these cases. There are some small numeric differences in terms of the 96 NxSxN penta-nucleotide substitution counts comparing to the signature training dataset, possibly due to different variant filtering strategies.

### Replication cohort

The whole-genome sequencing data generated within the CATCH study comprise 258 metastatic breast cancer samples. Tumor tissue and matched normal control sample for sequencing were obtained after receiving a written informed consent under an institutional review board-approved protocol, details can be found in the CATCH publication^40^.

In short, the whole-genome sequencing data from the replication cohort were processed by the DKFZ OTP pipeline^41^. The pipeline used BWA-MEM (v0.7.15) for alignment, biobambam (https://github.com/gt1/biobambam) for sorting and sambamba for duplication marking. The tumor-germline paired alignments were then used by DKFZ indel SNV callers for indel and SNV discovery^42^ (https://github.com/DKFZ-ODCF/SNVCallingWorkflow, https://github.com/DKFZ-ODCF/IndelCallingWorkflow).

### Somatic single nucleotide substitution variants(sSNV) artifacts removal

Somatic single nucleotide substitution variants (sSNV) artifacts were removed similar to the approach described a previous study^43^. Somatic single nucleotide variants present in over 1% of the samples in the local control list comprising 4879 WGS control samples from in-house cohorts were considered as technical artifacts and were removed from the driver analysis dataset.

To further exclude hotspots potentially due to sequencing artifacts, signatures that are potentially associated with sequencing artifacts were removed from the driver hotspots analysis^6^. For NxSxN-extended signatures, signatures found to be frequently associated with our in-house control list were removed.

### APOBEC substrate optimality analysis

APOBEC substrate optimality prediction was performed by ApoHP(initial1)^30^. The prediction was restricted to recurrent (n > 3) hotspots for tumors showing more than 5% APOBEC activity (n = 2636). Pearson correlation was calculated between log_2_ transformed substrate optimality and log_10_ transformed significance of APOBEC test from sigDriver.

### Signature exposures estimation

Five candidate assignment tools were compared for signatures assignment: sigProfiler (v2.5.14)^18^, Quadratic programming, YAPSA (1.16.0)^44^, sigfit-NMF and sigfit-Emu (2.0.0)^45^. Different mathematical methods are employed by each assignment method: sigProfiler uses constrained convex optimization, YAPSA uses linear combination decomposition, sigfit-NMF uses non-negative matrix factorization, sigfit-Emu uses expectation maximization, and quadratic programming is an in-house method using quadratic programming for signature exposures assignment. A signature benchmark in the translation table described in Supplementary Data 21 and Supplementary Fig. 10 showed sigProfiler showed the best performance among those. Therefore all signature exposure estimations were performed by sigProfiler^2^. Details of the benchmark were described in Supplementary Note 2, “Testing the assignment algorithms by known biological processes active in both contexts”.

### Sample QC for association tests

There are 121 specimens from the PCAWG cohort originating from different biological tumor specimens of the same patient, they were removed from the analysis (Supplementary Data 1). The unresolved relationship between these specimens can interfere the association test. For the somatic SNVs, an in-house blacklist on sequencing artifacts was used to filter the variants. After the removal of artifact SNVs, more than 38 million of SNVs remained for association tests.

### Genomic window pruning algorithm

The hotspot search is based on assumptions that many driver mutations form clusters on a functional domain in the genome. Some of the hotspots are in the non-coding regions and they could regulate one or more genes nearby. Region pruning in a given genomic window allows the test to focus on the most important sites and to disregard background mutations. A hotspot search is therefore designed to find mutation cluster(s) per predefined region, where some regions could carry multiple hotspots. The kernel regression method allows point mutations of different effect sizes in each tested region, the phenomenon is common in major oncogenes and tumor suppressors where more than one mutational process can be involved. However, a limited number of mixed effects can be modeled per region. The window pruning was performed by following procedure:

1. Restrict sSNVs to entities involved in each association test. In the normal mode, it is restricted to entities with at least 5% tumors positive for the signature, while the restriction is at 2% for the wide window mode.
2. Perform overlapping sliding window search per 30 bp at steps of 15 bp. Let a window *i* in tumor *t* be defined as *u*_it_ with *i* = 1,…, *I* and *t* = 1,…,*T*, where *I* is the total number of sliding windows and *T* is the total number of tumors. Let each sSNV *j* in tumor *t* be *sSNV*_jt_ with *j* = 1,…, *J*, where *J* is the total number of sSNVs.
  - Let the total number of somatic single nucleotide variations of tumor *t* be
    1 $${V}_{t}=\mathop{\sum }_{j=1}^{J}{{{{{{{\rm{sSNV}}}}}}}}_{{jt}}$$
  - sSNVs were binned per tumor *t* into window *i* and represented by *l*_it_. The number of variants *l*_it_ in window *u*_it_ is denoted by,
    2 $${l}_{{it}}=\mathop{\sum}\limits_{j\in {u}_{{it}}}{{{{{{{\rm{sSNV}}}}}}}}_{{jt}}$$
  - The per tumor weight *w*_*t*_ is defined by,
    3 $${w}_{t}=\frac{1}{{{\log }}_{2}{V}_{t}}$$
  - Compute the weighted mutational load *L* of the window *i* by,
    4 $${L}_{i}=\mathop{\sum }_{t=1}^{T}{w}_{t}{l}_{{it}}$$
3. Window *i* is defined as a hotspot when satisfying all the following conditions, with *L* presenting a vector containing all *L*_*i*_,
  5 $${L}_{i}\ge \frac{{\max }\;\left(L\right)}{2}$$
  6 $${L}_{i} > 3* {{{{{{\rm{mean}}}}}}}\,(L)$$
  7 $${L}_{i}\ge {{{{{{\rm{mean}}}}}}}\,\left(L\right)+{sd}\,\left(L\right)* 3$$

### Genome-wide association test

We performed a genome-wide search for putative drivers regardless of genomic and epigenomic annotations. For this purpose, sliding windows of 2 kb at steps of 1 kb over the whole genome were created. Windows with less than 6 somatic variants were discarded. Afterwards, each window undergoes pruning by the genomic window pruning algorithm. Remaining hotspot mutations were used in the following association test. The number of tumors tested is presented in Supplementary Data 5, 7, 12, and 13.

The sample size of this study provides the statistical power for testing association between somatic mutation burden of hotspots and signature exposures. In detail, the hotspot association test was performed per window after pruning. Association tests were performed on sSNVs of unknown effects, where the relationship with normalized exposures can be non-linear. To account for this, kernel regression was preferred over general linear models. The association tests were only performed when the minimum recurrence per site is > = 6 or the total number of variants within region is > = 10 given minimum recurrence > = 3. The association tests were corrected for confounders including tumor mutation load, entity and the sex of the patient. Each variant is weighted by $R\times {{{{{{\rm{median}}}}}}}\left(L\right)$, where *R* is the recurrence of the site and *L* is the somatic mutational load for tumor(s) carrying the variant. The regression model is given by:

8 $${{{{{{\rm{signature}}}}}}}{{{{{\rm{\_}}}}}}{{{{{{\rm{exposure}}}}}}} \sim \beta \,{{\bullet}\, {{{{{{\rm{Genotype}}}}}}}}_{{ij}}+{{{{{{\rm{entity}}}}}}}+{{{{{{\rm{nvar}}}}}}}+{{{{{{\rm{gender}}}}}}}$$

where $\beta$ was tested for significance in the test, ${{{{{{\rm{Genotype}}}}}}}$_*ij*_ is the genotype matrix of the tested genomic window where ${{{{{{\rm{Genotype}}}}}}}$_*ij*_ = 0 or 1, nvar refers to the mutational load of tumors, and *signature_exposure* refers to the normalized signature exposures of the tested mutational signature.

The association was performed by a rare-variant kernel regression association R-package SKAT(2.0.1)^17^ with the possibility of providing weight to each tested point mutation and detection of negative effects. The model SKAT-O was selected as an optimal method given its performance on mixed-effect^17^. The efficient resampling method provided by the package was used to calibrate the *p* values in each test performed^29^. The *p* values were corrected by Bonferroni correction considering the number of tests performed per signature.

Surrogate signatures were found across all three sets of tested mutational signatures (50 COSMIC SBS V3 signatures, 61 Penta-nucleotide signatures, 43 NxSxN-extended signatures). Surrogate signatures reflecting APOBEC activity can be found across all three set of signatures. Hotspots assigned to surrogate signatures of APOBEC activity (SBS2/13 and SBS-E2/E13) were in good agreement (>=78% overlap, Supplementary Fig. 7) and were combined for the analysis.

### Removal of signatures from candidate signatures related to driver mutations

The distributions of *p* values were evaluated by quantile-quantile plots (Supplementary Fig. 11). Some signatures have too many small *p* values which suggest higher false positive rate or poor model fitting. Signatures with many small *p* values (λ > 1.5) were removed from putative driver analysis (Supplementary Data 22).

### Enhancer and transcription start site (TSS) annotation using 18-state ChromHMM from 98 epigenomes

To annotate hotspots, each of them was intersected with 98 epigenomes from roadmap epigenetics^23^. For enhancers, the ratio between enhancer states (EnhG1, EnhG2, EnhA1, EnhA2) and background states (ChromHMM states 1–6, 12–14) were considered. If the ratio is more than 1:2, hotspots are annotated as enhancers. For transcription start site (TSS) annotations, a similar approach was taken using the ratio between TSS states (TssA, TssFlnk) and background states (ChromHMM states 5–6,7–15).

### Evaluating the significance explained by each somatically mutated site

The association tests report only the significantly associated regions. To further resolve the contribution of each somatic point mutation, the *p* values were perturbed by dropping one site at a time to evaluate the significance explained by each site. The significance explained by each somatic mutation was evaluated by the *P*_t_/min(*P*) where *P*_t_ is the perturbed *p* value after dropping the site and *P* is a vector of perturbed *p* values of all point mutations in the hotspot. The results of the perturbation can be visualized by a lollipop plot (Figs. S1, S2, S3).

### Genomic annotations and enrichment analysis

Each hotspot was annotated by ANNOVAR (2019Oct24)^46^ using refGene annotations on hg19. Only the top point mutations of the associated hotspots were considered for this analysis. The annotated coding and regulatory elements were taken together for enrichment analysis by the empirical *p* values distribution derived by permutation. The empirical *p* values were calculated by 100.000 rounds of permutation by simulating point mutations over the mappable genome (mappability > 25 at read length 100 bp)^47^. The mappable estimates were provided by umap (0.2.7.0)^47^, regions with mappability smaller than 0.25 were removed from the test. The permutation test is one-sided.

### Transcription factor binding sites annotation and enrichment analysis

Top point mutations from hotspots were annotated for transcription factor binding sites (TFBS) using TFBS clusters (V3) from ENCODE^31^. All recurrent sSNVs were annotated likewise for comparison. The enrichment of TFBS from each tested signature was performed by hypergeometric test.

### Known driver annotation and enrichment analysis

Known drivers were curated from drivers identified in 17 studies (Supplementary Data 6). As our method is using the information on mutational processes, we included the driver candidates removed due to a strong link to specific mutational processes in the PCAWG driver study and named this extended list “mutational processes-inclusive list of PCAWG candidate coding drivers”. In the list for driver genes curated from 17 studies we identified 802 unique driver genes after removing overlapping genes. To compute known drivers overlapping with COSMIC Cancer Gene Census (CGC)^25^, tier 1 cancer driver genes were taken for comparison, overlaps by gene names were presented in Supplementary Data 5. The enrichment of known drivers from each signature was performed by hypergeometric test on the basis of 21 thousand coding genes.

### Differential expression analysis

The differential gene expression analyses and differential transcript expression analysis were performed by DESeq2 (1.26.0). For cross entity analysis, entities without carriers of hotspot mutations or with less than 5% tumors positive for the mutational signature were removed from the test. For both cross entities analysis and single entity analysis, differential expression analysis was performed only when the test set had three or more hotspot-positive tumors.

The analysis was restricted to 1359 tumors where isoform counts from RNA-seq data provided by PCAWG were available. The differential expression analyses were performed on hotspots associated with mutational signatures. Tumors carrying at least one hotspot were contrasted with tumors without hotspot mutations. The differential gene and transcript expression analysis was performed under the following model:

9 $$y \sim {{{{{{\rm{genotype}}}}}}}+{{{{{{\rm{CNR}}}}}}}+{{{{{{\rm{entity}}}}}}}$$

CNR is the log_2_ copy number ratio between the copy number of the tested gene and the estimated ploidy. To assess the distribution of *p* values across differential gene expression tests, the quantile-quantile plot was presented as Supplementary Fig. 12.

### Identification of artifact signatures

To investigate signatures frequently associated with artifacts, two different variants sets were used for association. The artifacts list was defined by an in-house artifacts list of SNVs and Indels often found in the output of the DKFZ somatic mutational pipeline. The two variant sets tested are namely variants before artifacts filter and variants after artifacts filter. Independent association tests were performed on both variant sets and associated hotspots were further investigated. Each associated hotspot is further refined by perturbation to point mutation resolution and each of the most associated sites are compared with the artifacts list. When a signature is frequently associated with artifact sites, the signature is likely to be an artifact. Signatures frequently associated with artifacts were removed from the analysis.

### Prediction of differential transcription factor binding

Using DeepBind (0.11)^31^, the binding affinity changes of 515 human transcription factors caused by somatic mutations were evaluated. From each of the reported hotspots, only the top point mutation was taken for evaluation. Predictions were only performed when the transcription factor binding sites (TFBS) are active on the hotspot, indicated by TFBS clusters (V3) from ENCODE^31^. For each site, the FASTA sequence ±20 bp from the mutations were taken, the binding affinity of the mutant sequence and the wild-type sequence were compared across 515 human transcription factors. Differential binding sites were defined by the following conditions: (1) the wild-type sequence has >=0.6 probability to be bound by a transcription factor, (2) the binding probability of the mutated sequence drops by >=0.4

### Statistics

All tests in this study are two-sided unless otherwise mentioned. If no statistical method is provided the *p* values or adjusted *p* values are resulting from sigDriver. Analyses were performed with R (3.6.0), and visualized with ggplot2 (3.3.3).

### Reporting summary

Further information on research design is available in the Nature Research Reporting Summary linked to this article.

## Supplementary information

Supplementary Information

Supplementary Data

Data Source Tables

Reporting Summary

## Data Availability

Somatic variant calls from PCAWG and ICGC were obtained from the ICGC web portal (https://dcc.icgc.org/api/v1/download?fn=/PCAWG/consensus_snv_indel/final_consensus_passonly.snv_mnv_indel.icgc.public.maf.gz, s3://pcawg-tcga/consensus_snv_indel/final_consensus_passonly.snv_mnv_indel.tcga.controlled.maf.gz), the TCGA section of the dataset is under controlled access (https://dcc.icgc.org/releases/PCAWG/consensus_snv_indel), access can be requested through https://dbgap.ncbi.nlm.nih.gov/. Download details are provided at http://docs.icgc.org/pcawg/data/#download-from-pdc. Somatic variant calls from the pediatric cancer cohort were obtained from the R2 database (https://hgserver1.amc.nl/cgi-bin/r2/main.cgi?&dscope=DKFZ_PED&option=about_dscope). Tumors from the CATCH cohort were collected by National Center for Tumor Diseases (NCT) in Heidelberg and were processed by ODCF using DKFZ whole genome sequencing and transcriptome sequencing pipelines. Sequencing data for CATCH can be found at EGA under accession ID: EGAD00001007563. The CATCH dataset is under controlled access, please contact hipo_daco@dkfz-heidelberg.de to request for access permission. Datasets described specifically in this manuscript can be found in the Supplementary Data. Source data are provided with this paper.

## Code availability

The code of sigDriver for driver discovery and NxSxN-extended signature definitions are available at (https://github.com/wkljohn/sigDriver)^48^.
