# Supplementary material for: Association of mutation signature effectuating processes with mutation hotspots in driver genes and non-coding regions
Source: Nat Commun. 2022 Jan 10;13:178. doi: 10.1038/s41467-021-27792-6 (PMC8748499; doi:10.1038/s41467-021-27792-6)
Supplement: Supplementary file 1 — Supplementary Information [file 41467_2021_27792_MOESM1_ESM.pdf]

Supplementary Information

Table of Contents

Supplementary Notes 4

1. NxSxN-extended signatures — Signature extraction 4

Extracting mutational SNV signatures from NxSxN penta-nucleotide context 4

Merging of NxSxN-extended signatures 5

Uncovering the link between NxSxN-extended signatures and COSMIC SBS signatures V3.0 5

Comparisons with penta-nucleotide signatures 5

Novel signatures in the NxSxN-extended context 6

2. NxSxN-extended signatures — Signature applications 7

Combining NxSxN-extended signatures and COSMIC SBS signatures for prediction 7

Evaluating the reproducibility of signature discovery using sigProfiler 7

Evaluating the accuracy of exposures estimation by NxSxN-extended signatures 7

Landscape of NxSxN-extended signatures in tumor entities (Including PCAWG/Pediatric tumors) 8

Testing the assignment algorithms by known biological processes active in both contexts 8

3. Coding drivers associated with mutational signatures 10

Coding drivers on *IDH1* and *H3F3A* are associated with mutational signatures 10

4. Impacts of hotspots associated with mutational signatures 10

Expression impact of *RAD51B* hotspot mutations associated with APOBEC activity 10

NxSxN-extended signature hotspots impact on expression 10

Hotspots associated with mutational signatures as susceptible sites 11

SBS32/SBS37 hotspot mutations enriched on LINE elements 11

Enrichment of the AAAACTTA motif on signature SBS17 hotspots 11

Activation-induced cytidine deaminase (AID) hypermutation signatures (SBS9/SBS-E9/SBS84) identified hotspots of somatic hypermutation 11

Supplementary Methods 12

1. Variant sampling correction by mutational signatures 12

2. PCAWG mutational processes-inclusive driver candidates 12

3. Building translation table between NxSxN and trinucleotide context signatures 12

4. Evaluating the performance of signature assignment methods 13

5. Structural variants annotations 13

6. Topologically associating domain (TAD) boundaries annotations 13

7. Landscape of NxSxN-extended signatures in tumor entities 13

8. Evaluating signature SBS3 and signature SBS-E3 as the classifier of BRCA1/BRCA2 deficiencies 14

Supplementary Figures 15

Supplementary Figure 1. Graphical illustration of the *TERT* hotspot: 15

Supplementary Figure 2. Lollipop plots gallery for significant APOBEC hotspots 16

Supplementary Figure 3. the *MAPKAPK2* hotspot 21

Supplementary Figure 4. *DYRK3* expression in breast cancer liver metastasis (CATCH) grouped by *MAPKAPK2* hotspot mutation status. 22

Supplementary Figure 5. APOBEC signature and SBS39 signature exposures among PAM50 breast cancer subtypes. 23

Supplementary Figure 6. Venn diagram contrasting hotspots among 3 mutational signature sets 24

Supplementary Figure 7. Venn diagram of APOBEC signatures overlap 25

Supplementary Figure 8: Additional gene expression comparison of hotspot mutations. 26

Supplementary Figure 9. Lollipop gallery for hotspots associated with somatic hypermutation 27

Supplementary Figure 10. Translation table between NxSxN-extended signatures and COSMIC SBS signatures V3 29

Supplementary Figure 11. Quantile-Quantile plot of association tests from various mutational signatures 30

Supplementary Figure 12. Quantile-Quantile plot for differential gene expression tests using DESeq2 32

Supplementary Figure 13: Description of NxSxN-extended context and signatures 33

Supplementary Figure 14. Heatmap showing cosine similarity between signatures deciphered by signeR and sigProfiler 40

Supplementary Figure 15. Translation table between NxSxN-extended signatures and Pentanucleotide SBS signatures 41

Supplementary Figure 16. The landscape of NxSxN-extended signature exposures across tumor entities. 42

Supplementary Figure 17. The receiver operating characteristic curve (ROC) of describing the prediction performance of signature SBS-E3, signature SBS3 and the combined maximum. 43

Supplementary Figure 18: The *IDH1*, *H3F3A* and *PTEN* coding hotspot mutations 44

Supplementary Figure 19. Cosine similarity of simulated signature exposures and estimated exposures of SBS13 using penta-nucleotide signatures (PSBS) and NxSxN-extended signatures (SBS-E). 45

Supplementary Figure 20. *RAD51B* isoforms information and the hotspot 46

Supplementary Figure 21. Motif analysis of SBS17 hotspots 47

References 48

Supplementary Notes

1. NxSxN-extended signatures — Signature extraction

Extracting mutational SNV signatures from NxSxN penta-nucleotide context

The construction of NxSxN signatures utilize the context information “N”s around the substitution “S” and results in 96 classes of NxSxN penta-nucleotide profiles (Supplementary Figure 13a). The 96 classes of NxSxN-extended context have the same complexity to that of trinucleotide information (xNSNx), enabling the use of signature discovery tools designed for trinucleotide context. The profiles from 5070 tumors were analyzed by SigneR (v1.8.0). SigneR uses an empirical Bayesian treatment of the NMF model to compute the signature profiles[6].

This deciphering step included the signature discovery dataset as input. The sample set was further split into two groups 1) Non hypermutators (n=4389) and 2) Hypermutators (n=681), similar to the approach taken by PCAWG[1]. Samples with more than 20 thousand SNVs were allocated to the hypermutators group where the remaining in the non-hypermutators group.

To increase the sensitivity for entity specific signatures, independent decipher runs were performed on independent entities with more than 100 tumors.

In each of the decipher attempts, the optimal number of signatures were determined by the average silhouette width of a 96 classes x 5000 iterations matrix generated by signeR. The optimal number of signatures was determined by the biggest drop in average silhouette width S between S_n and S_{n+1}, where n is the targeted number of signatures to fit. Subsequently, an initial set of 41 signatures was selected from a range of signature sets generated by signeR (Supplementary Figure 13b).

To investigate if NxSxN signatures are stable across signature discovery tools, the signature discovery process was repeated using sigProfiler, another software for decomposing signatures. A comparison has been made between signeR and sigProfiler (Supplementary Figure 14). Since cosine similarities cannot be used for mapping NxSxN-extended signatures to COSMIC single base substitution (SBS) signatures, we propose multiple approaches (see below) to explore similarities between NxSxN-extended signatures and COSMIC SBS signatures. For example, the NxSxN-extended signature set consists of two APOBEC signatures which are known to have a motif flanking more than +/-2bp of the substitution. Some signatures can be linked with COSMIC SBS signatures while the remaining ones are potential new signatures, summarized in Supplementary Data 24.

Merging of NxSxN-extended signatures

Signature discovery on the NxSxN-extended context was performed per entity and on the full signature discovery dataset. For per entity discovery, deciphered signatures were merged with the main NxSxN-extended signatures definitions. Signatures of high cosine similarities (>= 0.9) were discarded, resulting in a single NxSxN-extended signature set.

Uncovering the link between NxSxN-extended signatures and COSMIC SBS signatures V3.0

As some mutational processes work beyond the trinucleotide context, a translation table was built to indicate the similarity between the two independently deciphered single base substitution signature sets (COSMIC SBS signature V3 and NxSxN-extended signature). The translation table is a conditional probability table built by over 52 million SNVs over 3813 tumors. The table shows that the NxSxN context contains surrogate signatures for UV light activity and APOBEC activity. For example, UV light signature SBS7a maps to signature SBS-E7A (Supplementary Figure 10). Some high confidence translation between uncharacterized COSMIC signatures encouraged further investigations. All the potential matches between COSMIC SBS signatures V3 and NxSxN-extended signatures are summarized in Supplementary Data 24. The translation table is also useful for benchmarking signature exposure assignment methods explained in the signature application section. Instead of using reproduction error for evaluation, the method uses the probabilistic mapping between surrogate signatures. The translation table was used as the major evidence to determine the mappings between the COSMIC SBS signatures and our NxSxN-extended signatures. Comparable signatures were assigned with the same signature number. For example, APOBEC signatures in the NxSxN-context are also signature 2 (SBS-E2) and signature 13, similar to the numbering system of the trinucleotide context.

Comparisons with penta-nucleotide signatures

Penta-nucleotide signatures were also suggested by the recent PCAWG investigation on mutational signatures [1]. To look for additional signature mappings, a translation table was also built between NxSxN-extended signatures and penta-nucleotide signatures. Additional mapping was observed between PSBS72 and SBS-E120/E43B (Supplementary Figure 15).

Novel signatures in the NxSxN-extended context

Of 41 proposed signatures in the NxSxN-extended signatures set, in-depth analysis was performed to compare their relevance with trinucleotide COSMIC SBS signatures and their properties across tumor entities.

By combining evidence from shared hotspots, signature translation table, and signature activity correlation, we were able to identify NxSxN-extended signatures novel to COSMIC SBS signatures (Supplementary Data 23). Some of the NxSxN-extended signatures reported most novel hotspots such as SBS-E101 and SBS-E102, hotspots are missing from COSMIC signatures analysis.

The signature translation table indicated SBS12 can be further split into SBS-E12A, SBS-E12B, and SBS-E110 in the NxSxN-extended context (Supplementary Figure 10), where only SBS-E12A is prominent in liver cancer. Signature SBS-E12B and SBS-E110 are commonly found in other tumor types (Supplementary Figure 16).

2. NxSxN-extended signatures — Signature applications

Combining NxSxN-extended signatures and COSMIC SBS signatures for prediction

Among all NxSxN-extended signatures, the signature SBS-E3 was also found to be correlated with the BRCA deficiency of breast tumors. This enables two independent measurements of BRCAness on the same set of SNVs, by COSMIC signature SBS3 and also by NxSxN-extended signature SBS-E3. By taking a similar approach to HRDetect[2], the sensitivity of measurement could be improved by combining both signatures. Supplementary Figure 17 shows the receiver operating characteristic curve (ROC) for predicting BRCA1/2 status in the 560 Stratton breast cancer cases, using the formula:

BRCA1 or BRCA2 deficiency ~ max(Absolute_Exposure$_{SBS3}$, Absolute_Exposure$_{EX3}$)

The *BRCA1/BRCA2* deficiency status of each tumor is defined by the biallelic inactivation of the *BRCA1* or *BRCA2* gene. The biallelic inactivation status considers germline or somatic mutations in the categories of single base substitution, copy number loss of the wild type allele or loss of heterozygosity. The increased sensitivity of *BRCA1/2* detection using NxSxN-extended signature SBS-E3 potentially improve models for predicting homologous recombination deficiencies in tumor.

Evaluating the reproducibility of signature discovery using sigProfiler

Using the same criteria for choosing optimal number of signatures, we found 23 signatures using sigProfiler, compared to 35 from signeR using the non-hypermutated tumors in the discovery cohort. Deciphering of signatures was only attempted on the non-hypermutator set (n=4389) using sigProfiler. For the sake of comparison, all 41 signatures in the extended signatures were compared to 23 sigProfiler signatures. Using measurements of cosine-similarity, more than 60% of the signatures can be reproduced with unique match across two deciphering algorithms (Supplementary Figure 14).

Evaluating the accuracy of exposures estimation by NxSxN-extended signatures

To contrast the performance of penta-nucleotide signatures and NxSxN-extended signatures. Six simulations were performed using eight signatures which are present in both trinucleotide context and NxSxN-context (SBS2, SBS7a, SBS8, SBS12, SBS13, SBS17b, SBS22, SBS38). To assess the performance the two signature contexts given a signature utilizing NxSxN-context. The signature of interest was set to the APOBEC signature (SBS13), where 50% of noise was added to the trinucleotide context and the NxSxN-context remain unchanged. The signature definition and the respective signature exposures were estimated by SignatureAnalyzer[3]. The cosine similarity of simulated exposures of the modified APOBEC signature (SBS13) was compared between two signature contexts (penta-nucleotide signatures vs NxSxN-extended signatures), where NxSxN-extended signatures showed superior

performance in the simulation (Supplementary Figure 19). The run-time is about 12 times faster on NxSxN-extended signatures than penta-nucleotide signatures. The gain of accuracy and performance using NxSxN-extended signatures is likely due to the lower complexity of 96-classes of substitutions when compared with 1536-classes in the penta-nucleotide signatures.

Landscape of NxSxN-extended signatures in tumor entities (Including PCAWG/Pediatric tumors)

A tumor entity versus median signature exposures plot was built to indicate the enrichment of certain signatures in different tumor types (Supplementary Figure 16). By combining with the NxSxN-extended signature to COSMIC SBS signatures translation table, potential novel signatures are summarized in supplementary data 23.

Signature SBS-E3 is likely to indicate defective homologous recombination similar to SBS3, but with a higher number of entities observing the signature. Signature SBS-E3 was also shown to be more powerful in finding major coding drivers like *PIK3CA* and *TP53*. Signature SBS-E2/4B are APOBEC activity signatures, where their relevance was shown by correlation and signature deciphering by selected APOBEC positive cases.

There are artifacts signatures related to the sequencing or variants callers. For example, signature SBS-E113 is likely to be related to the behaviors of the DKFZ calling pipeline where the signature activity disappeared after filtering of somatic variants by an in-house blacklist for artifacts.

Testing the assignment algorithms by known biological processes active in both contexts

Using the translation table in Supplementary Figure 10, the performance of assignment algorithms can be assessed. The comparison employed conditional probabilities of SNVs assigned to surrogate signatures of a single mutational process. The performance of various signature assignment tools is presented in supplementary data 21 using surrogate signatures of APOBEC activity and UV exposures. The higher the conditional probability, the more SNVs have been assigned to the surrogate signatures of interest, for example SBS7a versus SBS-E7A. The ideal tool for mutational signature assignment should always assign SNVs caused by APOBEC mutagenesis to surrogate signatures of APOBEC (conditional probability = 1). Exposures of split signatures measuring the same mutational processes were summed for the comparison, thus exposures of SBS2/SBS13 and SBS-E2/E13 are summed for the comparison of APOBEC signatures.

Five signature assignment algorithms were tested: sigProfiler, Quadratic programming, YAPSA, sigfit-NMF and sigfit-Emu. sigProfiler is the best performing algorithm among them (Supplementary Data 21).

3. Coding drivers associated with mutational signatures

Coding drivers on *IDH1* and *H3F3A* are associated with mutational signatures

There are also well-established coding mutations in brain tumors identified by our method. The hotspot presented in Supplementary Figure 18 correspond to *IDH1* R132H mutation associated with SBS12(***p-adj*** **<** $\mathbf{8.85x10^{-13}}$) and SBS-E110(***p-adj*** **<** $\mathbf{4.76x10^{-9}}$), mostly in brain tumors . *H3F3A* K28M mutations are associated with a glioblastomas enriched for tumors with higher SBS1 exposure (**p-adj** **<** $\mathbf{4.14x10^{-8}}$), potentially due to higher age of *H3F3A* driver mutation carriers (r=0.60, CNS-GBM).

4. Impacts of hotspots associated with mutational signatures

Expression impact of *RAD51B* hotspot mutations associated with APOBEC activity

A hotspot was found on *RAD51B* in association with APOBEC signatures, and it is present across multiple tumor entities. The hotspot is located in the last intron of the longest *RAD51B* isoform, characterized by two point-mutations at chr14:69134626/69134629 (Supplementary Figure 20). Using the isoform expression estimates from PCAWG, differential transcript expression analysis was observed on two isoforms (Ensemble transcript ID: ENST00000488612, ENST00000390683) but in opposite directions in bladder cancer (ENST00000488612, upregulated $p < 0.003$, and ENST00000390683, downregulated, $p < 4.34x10^{-16}$, Supplementary Data 25). Meanwhile, there are no significant changes on the main isoform expression of *RAD51B* (Transcript ID: ENST00000487270). Using ChromHMM 18-states annotation from 98 epigenomes[23], the hotspot was found to be within an active enhancer in 27 epigenomes. As an important player in the DNA damage response pathways, the deregulation of *RAD51B* by APOBEC-mutagenesis can impact genome stability in tumors.

NxSxN-extended signature hotspots impact on expression

Gene expression and transcript expression analysis were performed when the entity has 3 or more hotspot-positive tumors (Supplementary Data 12). There are 16 of 49 NxSxN-extended signature hotspots linked to gene expression changes and 19 of 49 hotspots linked to differential transcript expression changes. Nine of the sixteen gene expression changes are overlapping with expression analysis of COSMIC SBS signature and are otherwise novel.

Hotspots associated with mutational signatures as susceptible sites

Hotspots from signatures SBS32 and SBS37 are not enriched for regulatory and coding elements. However, the number of hotspots associated with these signatures are high (>=20). By considering these hotspots as susceptible sites for mutational processes (Supplementary Data 4), motif analyses and exploratory analysis on genomic elements became possible.

SBS32/SBS37 hotspot mutations enriched on LINE elements

Hotspots from two uncharacterized signatures, SBS32 and SBS37 are enriched on line elements, comprising 88% (93 of 106) of the hotspots. This suggested a potential link to transposable elements. Both signatures SBS32 and SBS37 show similar prevalence on prostate adenocarcinoma and both signatures are likely to be related to the same mutational process.

Enrichment of the AAAACTTA motif on signature SBS17 hotspots

Signature SBS17 is a poorly characterized signature. Our method revealed large number of hotspots associated with the signature. Those hotspots are not enriched for regulatory elements or coding elements. HOMER *de novo* motif search was performed on the proximal flanking region of SBS17 hotspots (+/- 7b.p.), the analysis revealed enrichment of "AAAACTTA" motif on the hotspot ($p<1\times10^{-136}$,38% of hotspots, Supplementary Figure 21). The substitution is most common on the fifth base of the "AAAACTTA" motif. With the most common T>G and T>C substitution types in SBS17, the AAAACTTA motif became AAAAC[C>G]TA or AAAAC[T>C]TA. However, the mutational process behind the motif usage is poorly understood.

Activation-induced cytidine deaminase (AID) hypermutation signatures (SBS9/SBS-E9/SBS84) identified hotspots of somatic hypermutation

The associated hotspots from **A**ctivation-**i**nduced cytidine **d**eaminase (AID) hypermutation signatures (SBS9/SBS-E9/SBS84) are summarized in Supplementary Data 26. Large number of the hotspots overlap with known recurrent targets of somatic hypermutation in lymphoma [4]. The findings are consistent with our knowledge on the relationship between somatic hypermutation with polymerase eta signatures (SBS9/SBS-E9) and AID signatures (SBS84). Hotspots associated with somatic hypermutation are enriched on regulatory elements and some hotspots are novel.

Supplementary Methods

1. Variant sampling correction by mutational signatures

Different mutational processes target cancer genomes in different ways. Some attacks particular DNA bases on a motif, such as APOBEC mutagenesis. Some create a wide-spanning cluster of variants on the targeted region, such as somatic hypermutation. Our bin-pruning algorithm that aims to focus on most actively mutated part of the window inevitably introduce unevenness on sampling across mutational processes. To overcome this, the variant sampling correction routine of sigDriver performs a preparation pass on all samples to estimate how many genomic windows contain variants from each mutational signature after window pruning. The proportion of signature positive windows from each mutation signature is contrasted with the proportion of input samples positive for the signature, where they were considered respectively as observed and expected signature positivity in sampling windows. Probabilistic sub-sampling is performed to minimize difference in observed and the expected signature positivity in windows. The correction is performed on signatures except the signature of interest. The corrected variants in windows are subsequently used for association analysis.

2. PCAWG mutational processes-inclusive driver candidates

To enable reassessment of signature related candidate drivers from PCAWG. We retrieved coding and non-coding point mutations from the supplementary table 4 and 5 reported by PCAWG study of drivers[5]. These lists were produced by applying the false discovery rate filter (q < 0.1), mappability filter, < 3 mutations filter, < 3 patients filter, the manual review filter, and excluding small RNA and miRNA targets. The resulting lists consist 188 unique regions in the non-coding category, and 183 unique genes in the protein-coding category, which are considered as known drivers.

3. Building translation table between NxSxN and trinucleotide context signatures

The building of the translation table relies on the exposure of the NxSxN-extended signatures, exposure of the COSMIC SBS signatures V3 and the context information of each analyzed SNVs. For each of the included samples an S x C we built a matrix denoted by W_{sc}. S denotes the SBS signatures and C is the NxSxN-extended signatures. W_{sc} is a matrix of counts, counting the number of times where max(p_{si}) = max(p_{ci}) . The vector of SNVs is denoted by V, total number of variants per case denoted by v, E_s denotes the exposure of SBS signature S , formula for p_s are as follows:

$$p_{ci} = P(V_i|E_c), 1 < i < v$$

$$p_{si} = P(V_i|E_s), 1 < i < v$$

After computation of all n samples in cohort, the average probability of an SNV assigned to signature S which was also assigned to signature C becomes A_{sc}, which is computed by

$$A_{sc} = \frac{\sum_{i=0}^{n} \left(\frac{W_{sci}}{\sum W_{sci}} \right)}{n}$$

4. Evaluating the performance of signature assignment methods

Their performance was evaluated by their assignment stability to selected signatures: APOBEC and UV-light exposures. The stability was indicated by the value A_{sc} of the translation table, where s and c are the APOBEC/UV signature in the NxSxN-extended context and in the original context. The values were used to rank 5 of the assignment tools on 2 mutational processes. An overall rank was produced to evaluate the performance of each tool. sigProfiler achieved best performance in the benchmark.

5. Structural variants annotations

For interpretation of hotspots together with structural variants (SVs), structural variants within 200kb from hotspots were annotated. This analysis is available only for tumors analyzed in PCAWG.

6. Topologically associating domain (TAD) boundaries annotations

TAD boundaries were retrieved from a Hi-C study on Breast Cancer [6]. The study used an MCF-7 cell line to derive TAD boundaries. Given the relevance of the hotspots of interest in breast cancer, genes within the same TAD boundary were tested in the expression analysis.

7. Landscape of NxSxN-extended signatures in tumor entities

To investigate entities prevalence of NxSxN-extended, the signature training cohort was divided into 38 tumor entities. Signature exposures per entity is presented in Supplementary Figure 16, the median of normalized exposures is indicated by the color of the circles. The threshold for positive signature

attribution per tumor is 5%, where the proportion of positive tumor per entity was depicted by the size of the circle.

8. Evaluating signature SBS3 and signature SBS-E3 as the classifier of BRCA1/BRCA2 deficiencies

The *BRCA1/BRCA2* germline status were retrieved from the HRDetect dataset of 560 breast cancer patients[30]. The donor IDs were combined with the penta-nucleotide context information provided by the mutational signatures study of PCAWG (Synapse:syn11804040). SigProfiler was used to estimate the signature exposure of two signature sets: the COSMIC SBS signature V3, and the NxSxN-extended signature. The $\log_2$ absolute exposure of the corresponding HR deficiency signatures were combined with the *BRCA1/2* status for the receiver operating characteristic curve analysis (ROC). In addition to the signature exposure of the two HR deficient signatures, the maximum of the two were also used for prediction:

$$SBS3\_EX3\_Combined\_max = \max(\mathbf{\log 2}(\boldsymbol{AbsoluteExposure_{SBS3}}), \mathbf{\log 2}(\boldsymbol{AbsoluteExposure_{EX3}}))$$

The area under curve (AUC) was used in the ROC plot to evaluate the predictive performance of the three signature exposure vectors.

Supplementary Figures

Supplementary Figure 1. Graphical illustration of the *TERT* hotspot:

On the *TERT* hotspot, contributing SNVs at chr5:1295228 are characterized by C>T mutations, a characteristic substitution of spontaneous deamination (SBS1).

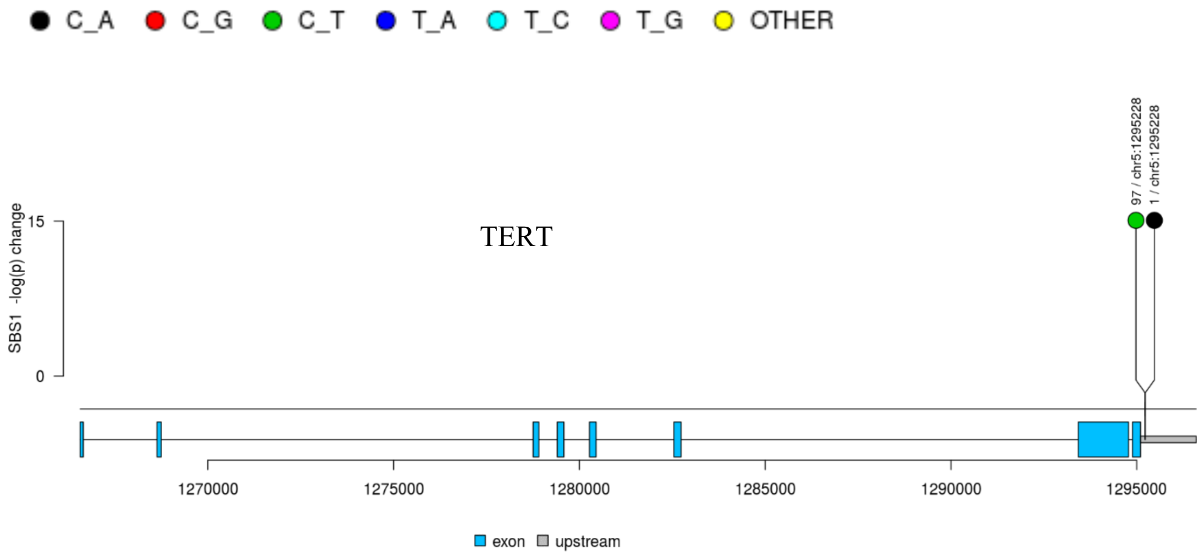

Supplementary Figure 2. Lollipop plots gallery for significant APOBEC hotspots

PIK3CA

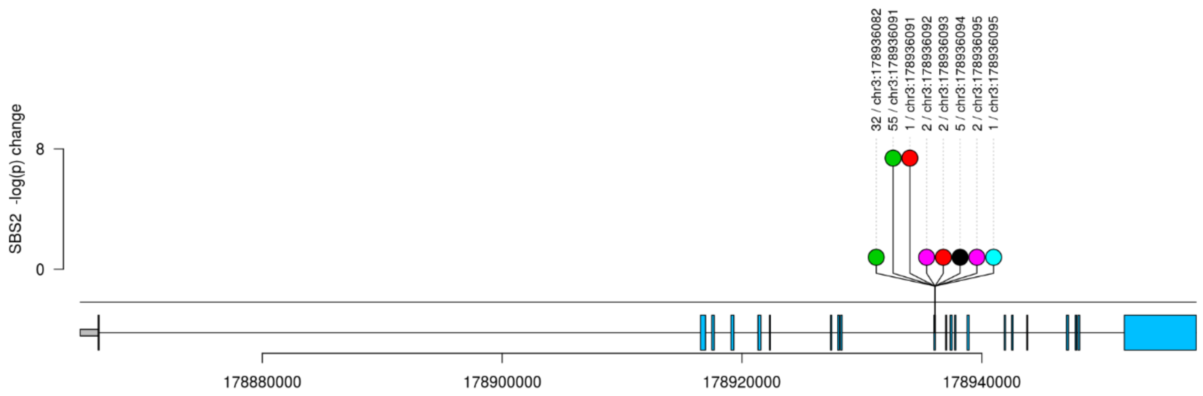

ADGRG6

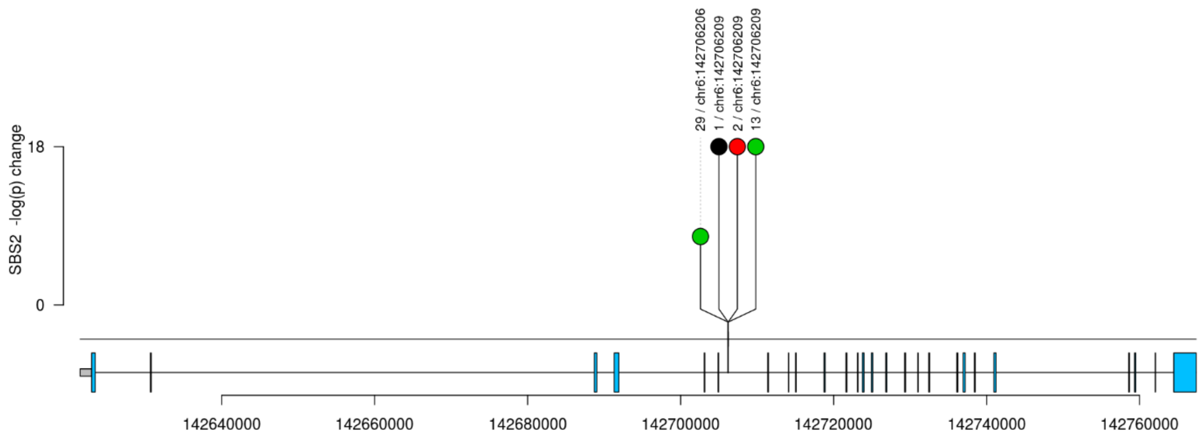

PLEKHS1

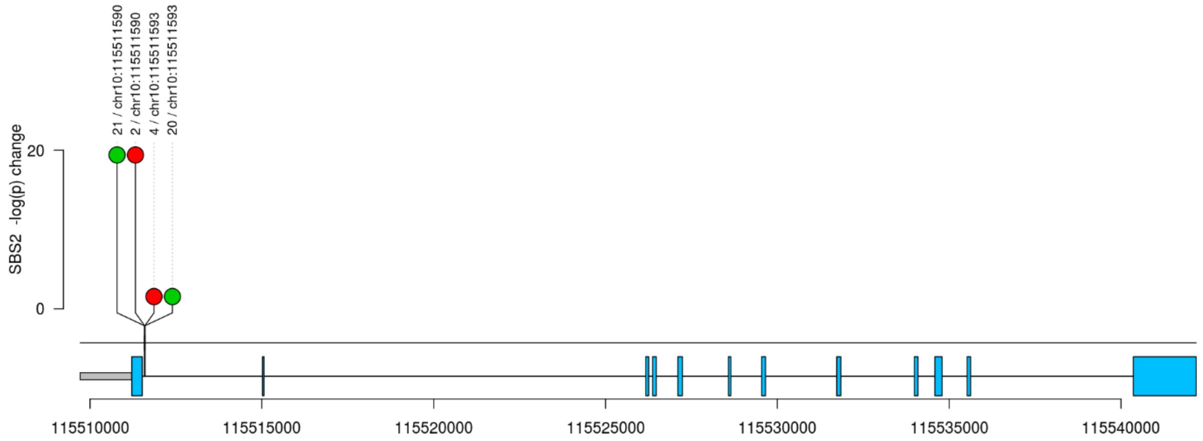

MAPKAPK2

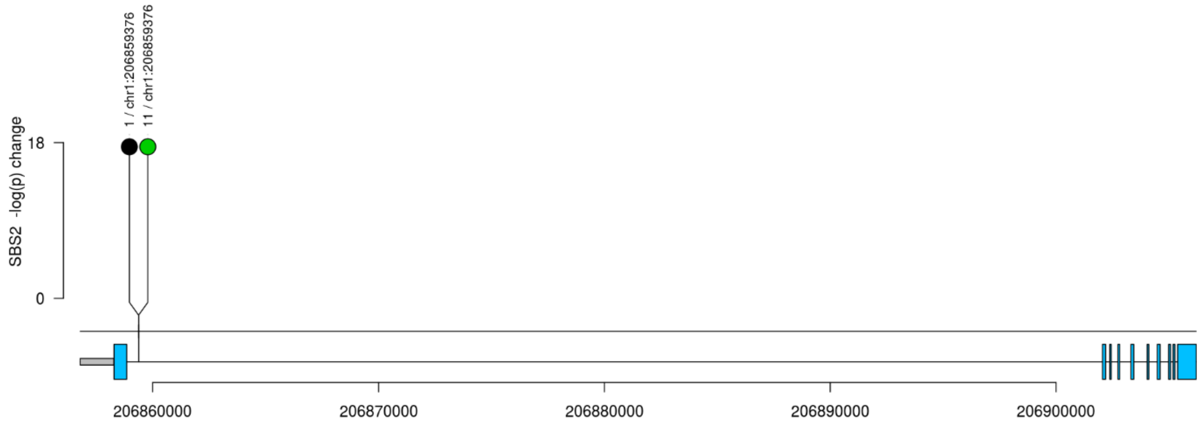

LEPROTL1

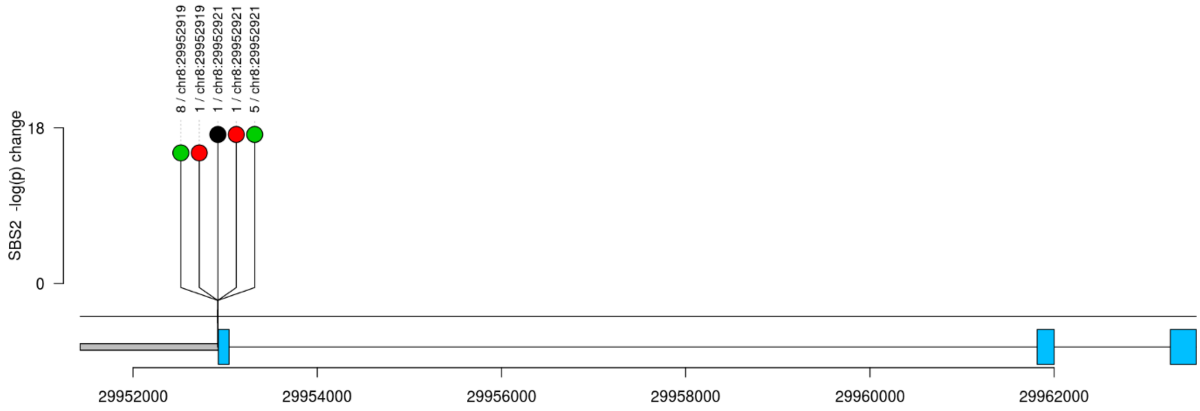

TBC1D12

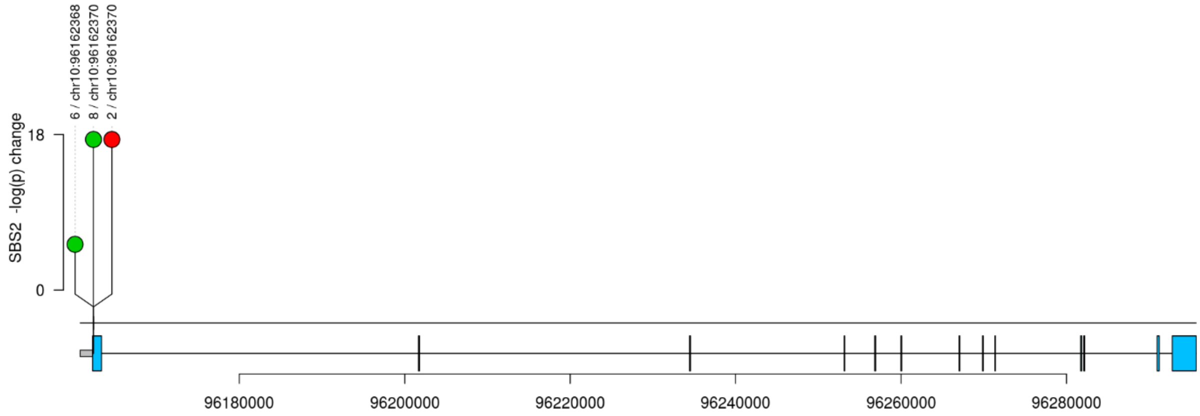

ADAMTS2

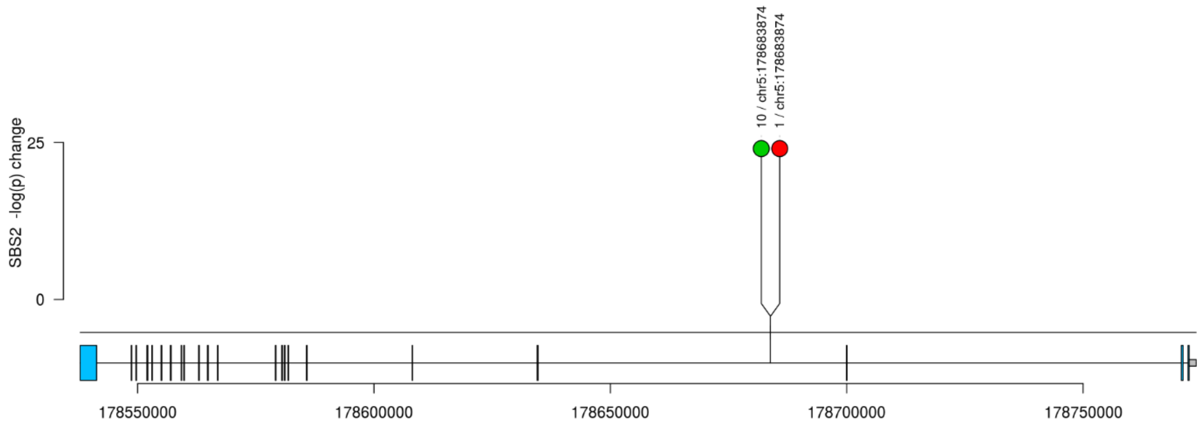

NTRK2

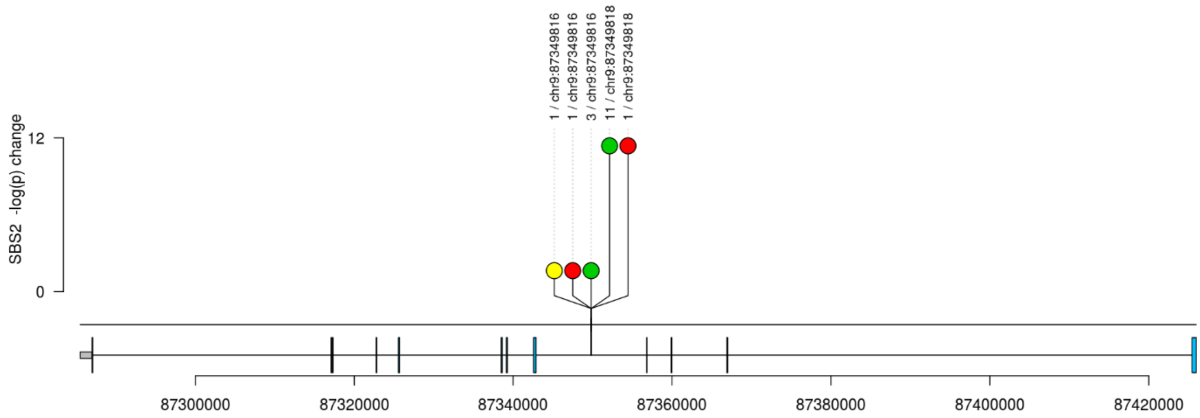

THOC2

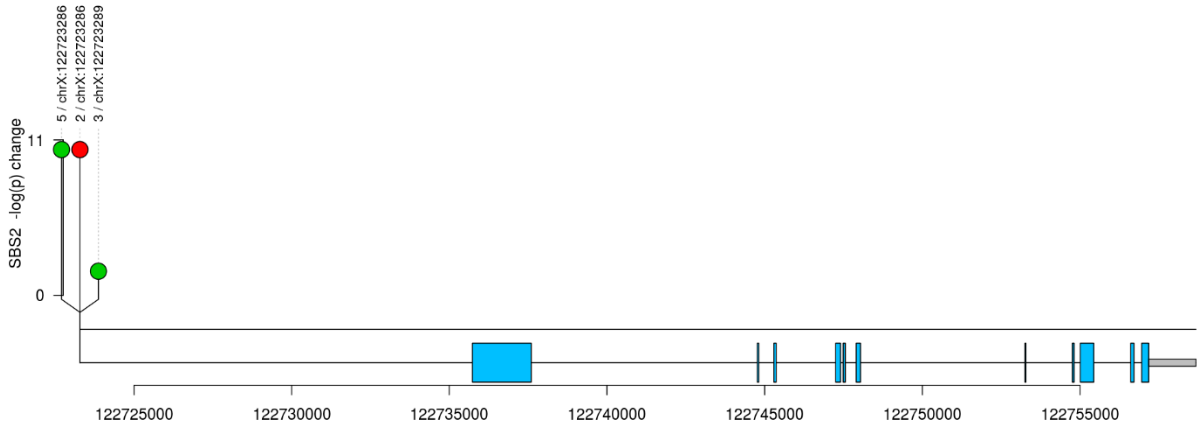

ANKRD53

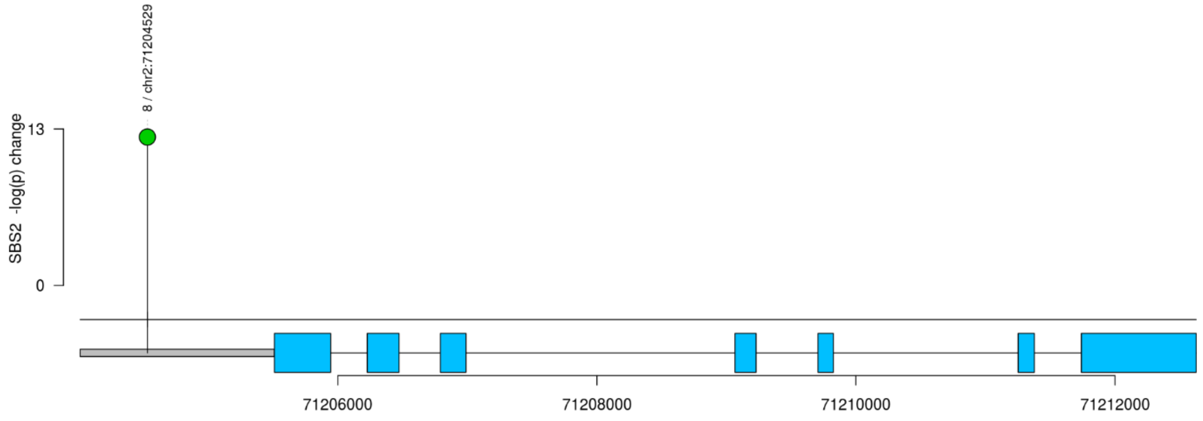

RAD51B

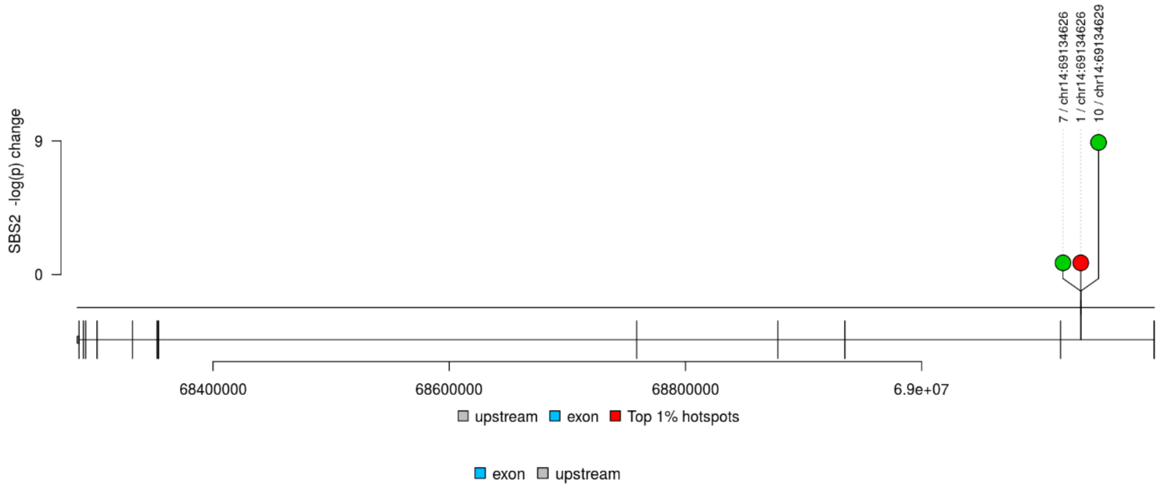

LINC02237

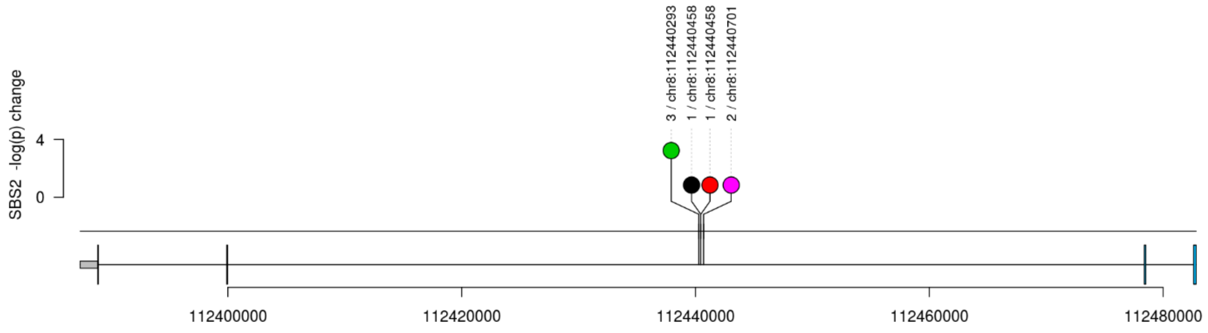

STAG1

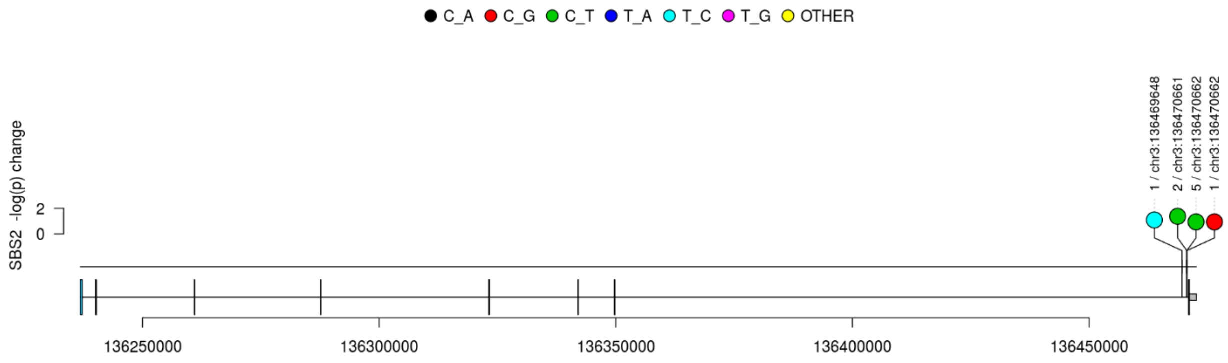

Supplementary Figure 3. the *MAPKAPK2* hotspot

The lollipop illustration of the *MAPKAPK2* hotspot, (b) the ChromHMM annotation of the hotspot from Roadmap epigenomics, (c) The ZBTB33 motif and the substituted base

(a) The hotspot mutation on intron 1 of *MAPKAPK2*

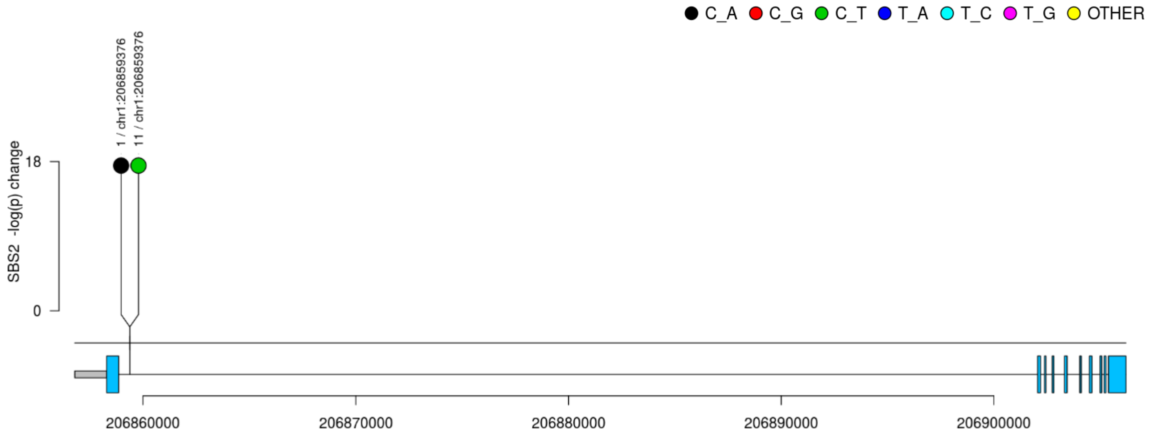

(b) The *MAPKAPK2* transcription start site information from 18-state ChromHMM model of 98-epigenomes, yellow line indicates the hotspot

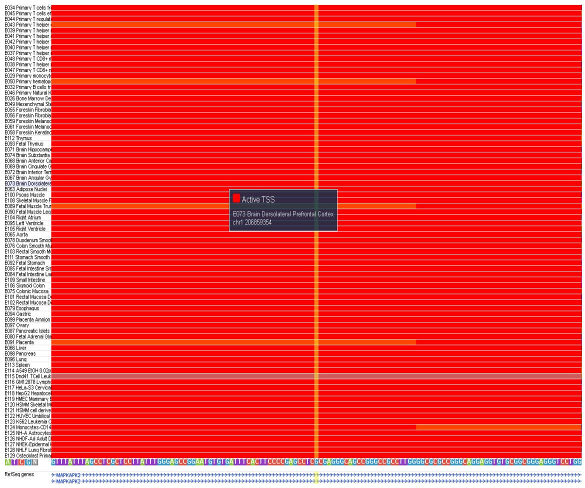

(c) The ZBTB33 core motif and red box indicated the substitution

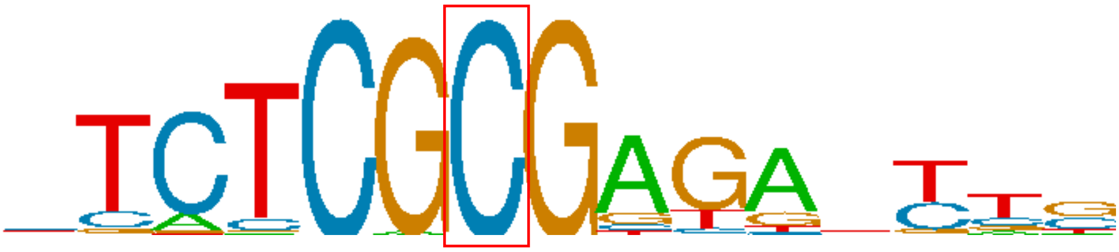

Supplementary Figure 4. *DYRK3* expression in breast cancer liver metastasis (CATCH) grouped by *MAPKAPK2* hotspot mutation status.

Significance and log2 fold changes using a two-sided test were provided by DESeq2 ($p < 0.003$). The boxes indicate the 25th and 75th percentiles with the median highlighted by a black line, whiskers extend to 1.5 times the interquartile range from the 25th and 75th percentiles, and polygons represent density estimates of data. Individual data points are presented as dots.

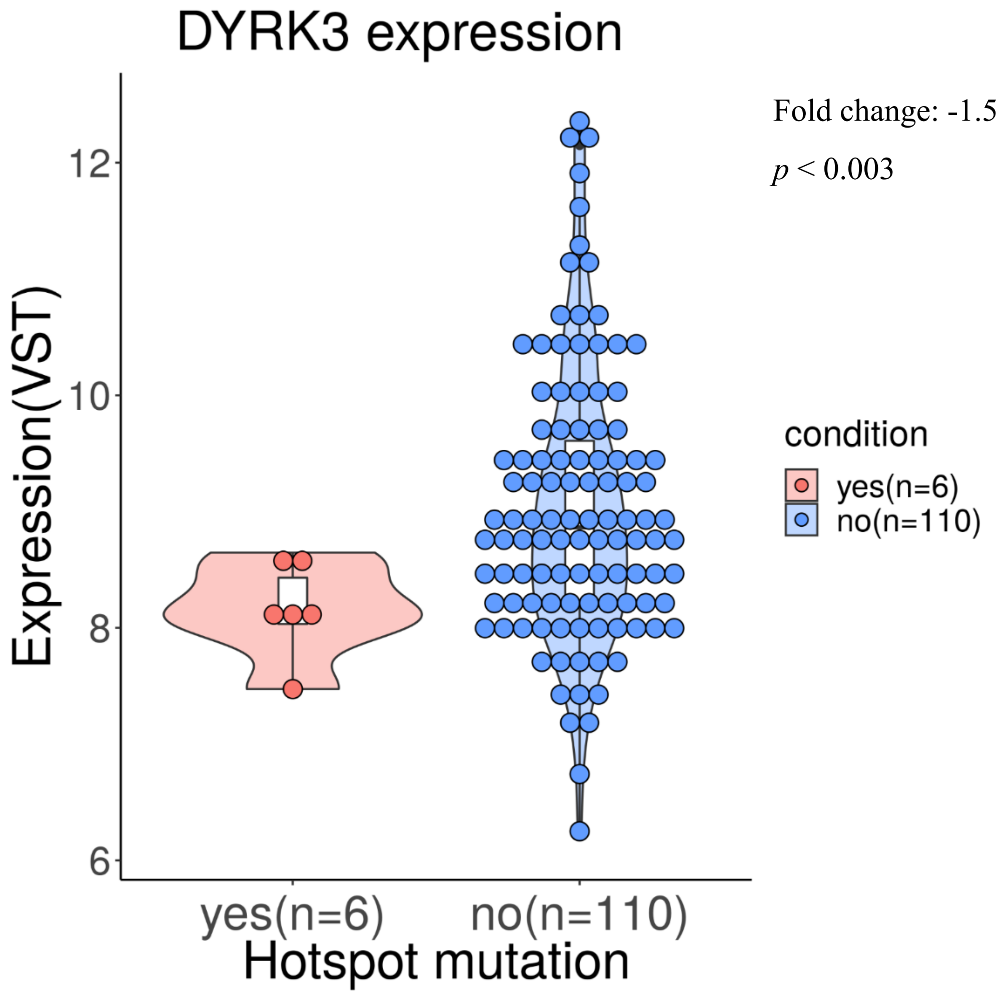

Supplementary Figure 5. APOBEC signature and SBS39 signature exposures among PAM50 breast cancer subtypes.

The boxes indicate the 25th and 75th percentiles with the median highlighted by a black line, whiskers extend to 1.5 times the interquartile range from the 25th and 75th percentiles, outliers are presented as individual dots. Differences in the normalized exposures were tested for significance by a two-sided Wilcoxon test (Basal vs Her2: p-value = 0.008561;Basal vs LumA: p-value = 0.004667;Basal vs LumB: p-value = 0.0008037). The significance level indicated by *** and ** are q < 0.001 and q < 0.01 respectively.

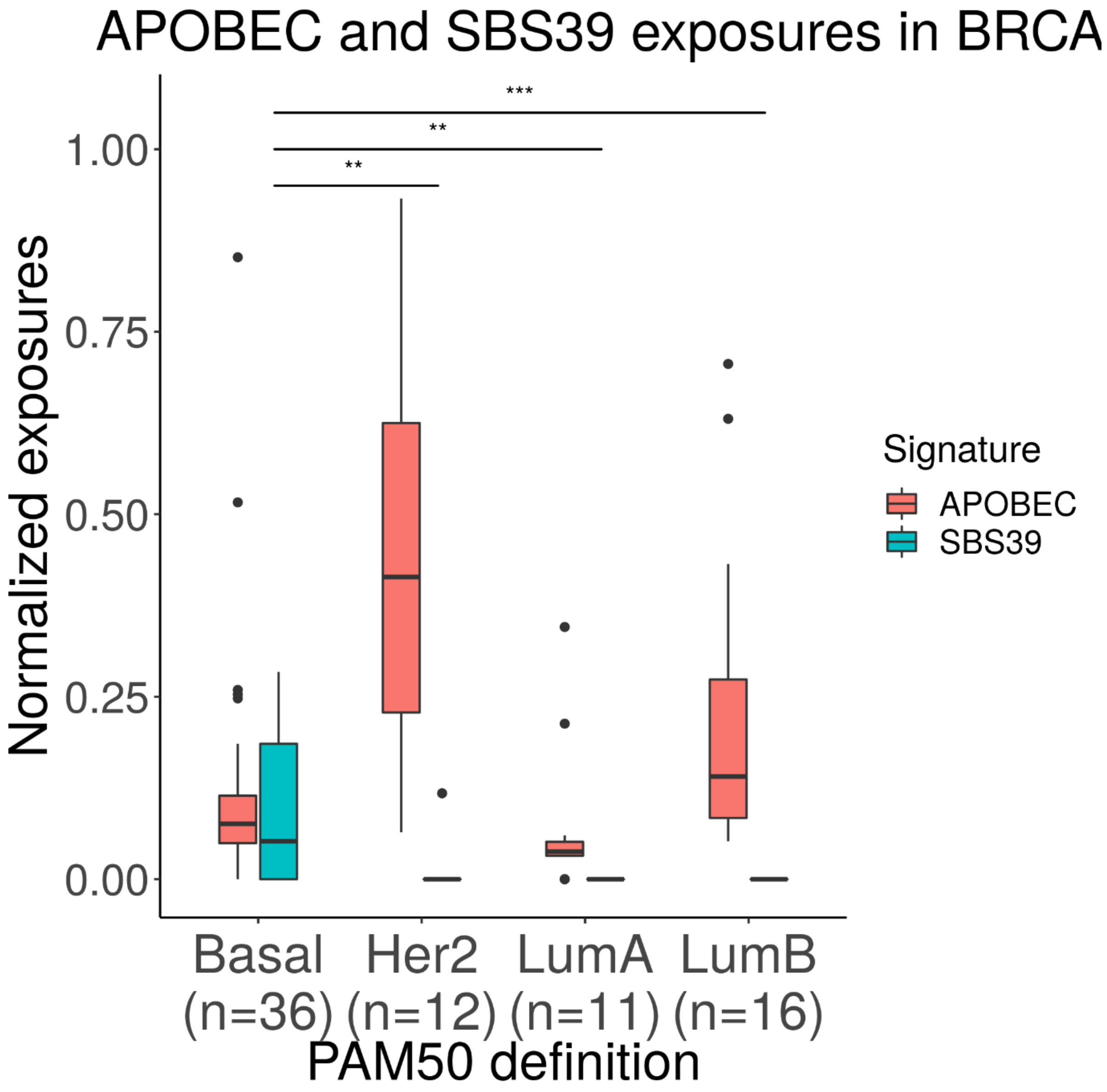

Supplementary Figure 6. Venn diagram contrasting hotspots among 3 mutational signature sets

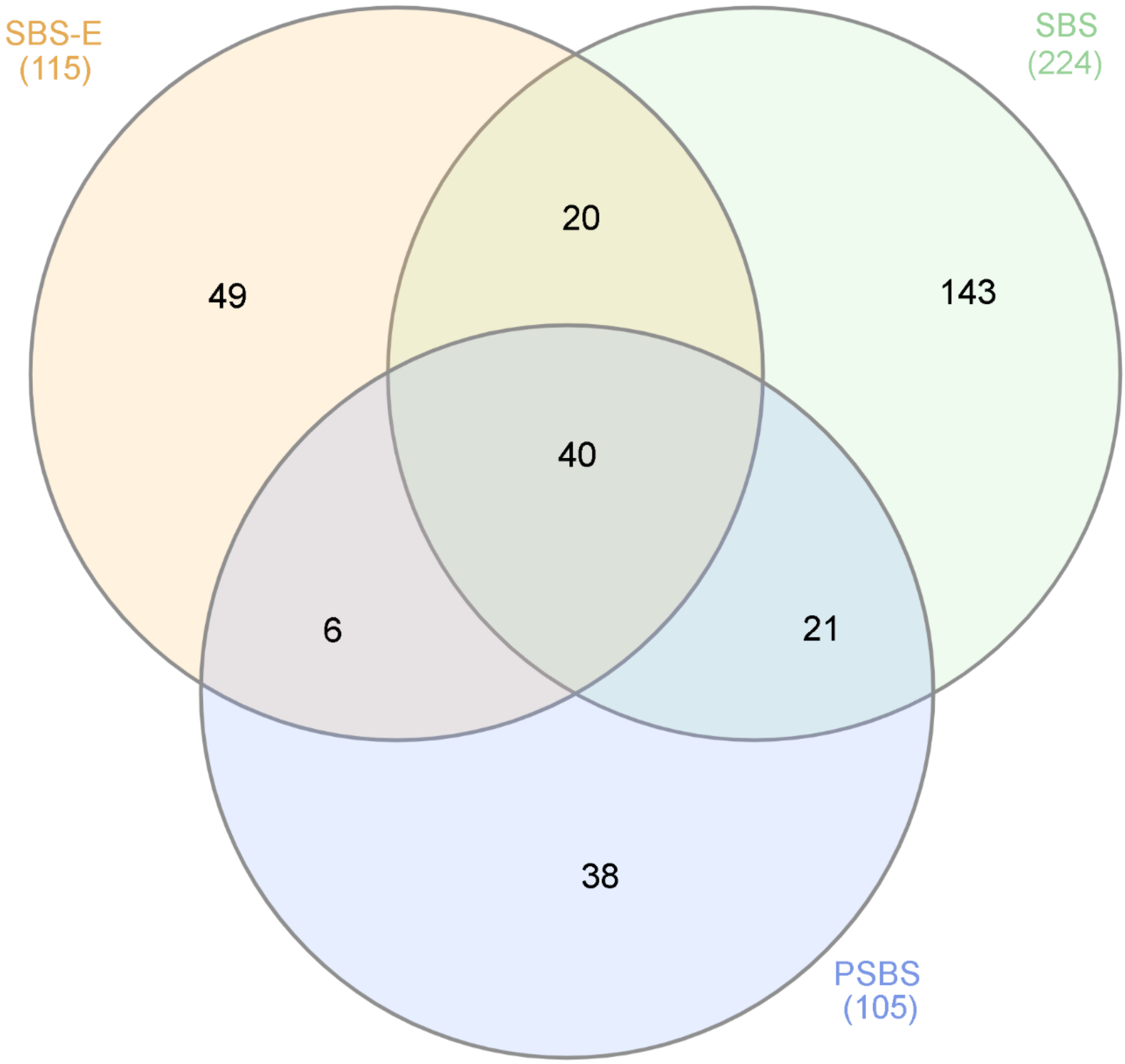

Supplementary Figure 7. Venn diagram of APOBEC signatures overlap

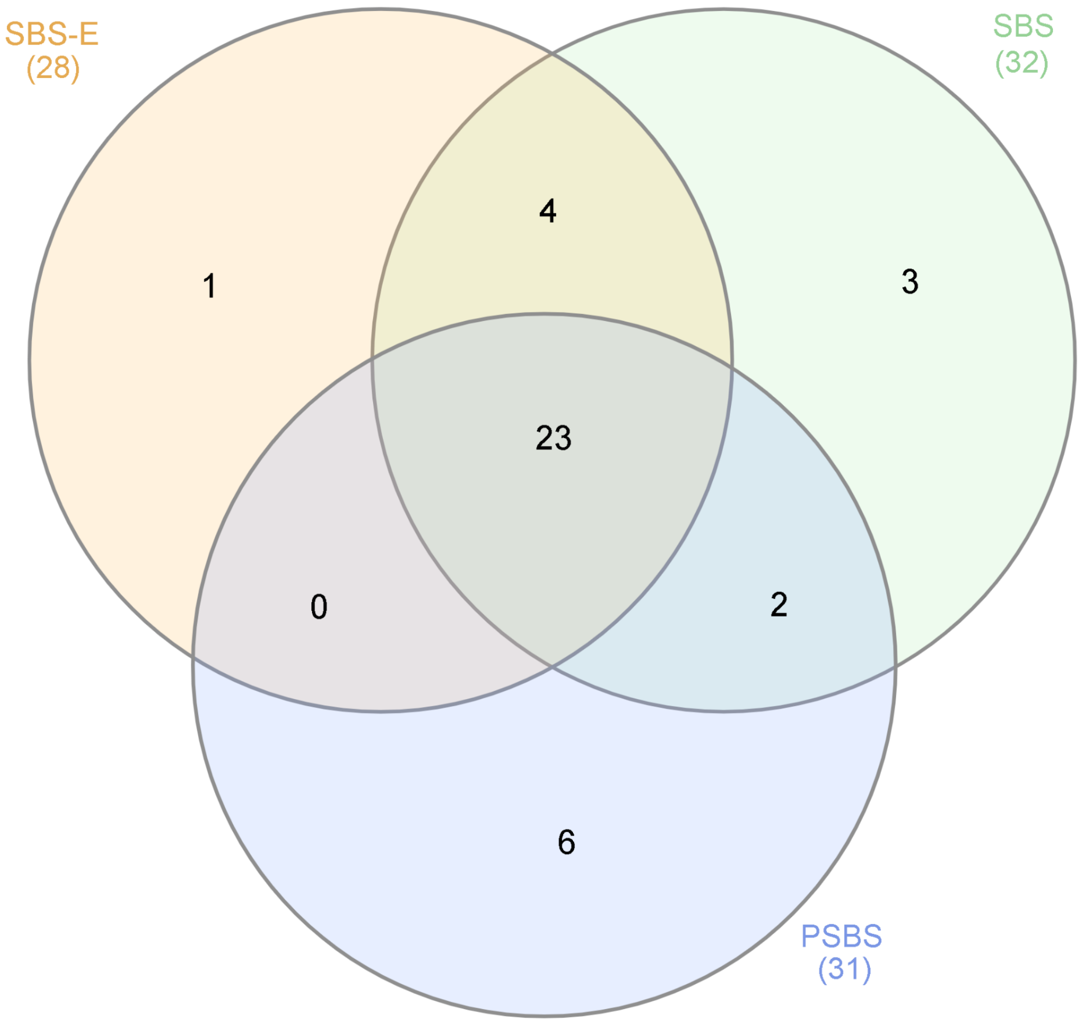

Supplementary Figure 8: Additional gene expression comparison of hotspot mutations.

Significance and log2 fold changes for two-sided tests were provided by DESeq2. P-values were FDR corrected by the total number of expression tests performed on the COSMIC SBS signature set, indicated by the q-values (Supplementary Data 16) (*LEPROTL1* q<0.82, *HAUS1* q< 0.59, *IGLL5* q<0.47). The boxes indicate the 25th and 75th percentiles with the median highlighted by a black line, whiskers extend to 1.5 times the interquartile range from the 25th and 75th percentiles, and polygons represent density estimates of data. Individual data points are presented as dots.

(a) APOBEC activity (Bladder-TCC)

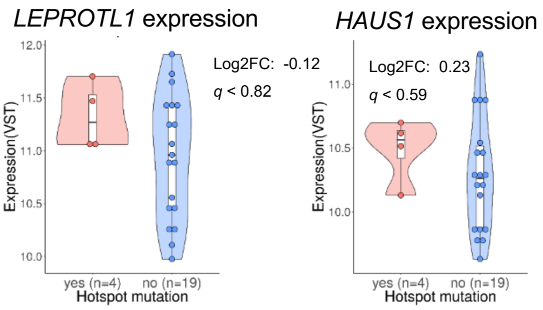

(b) Somatic hypermutation hotspots

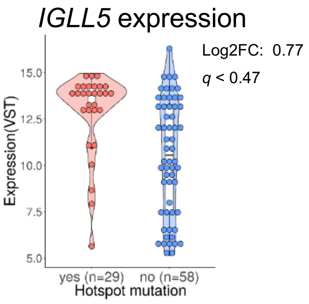

Supplementary Figure 9. Lollipop gallery for hotspots associated with somatic hypermutation

IGHJ6:

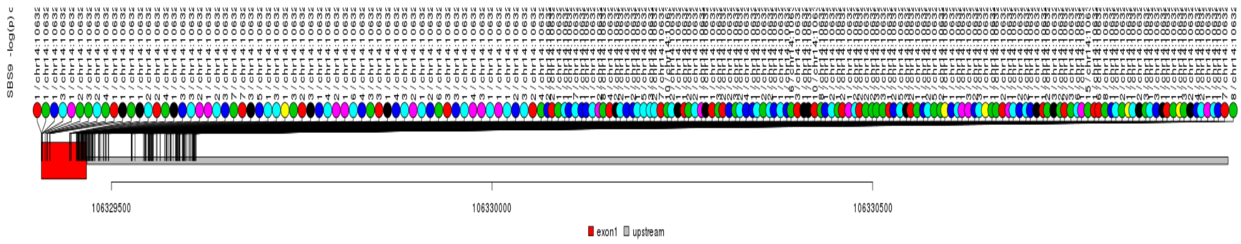

CXCR4:

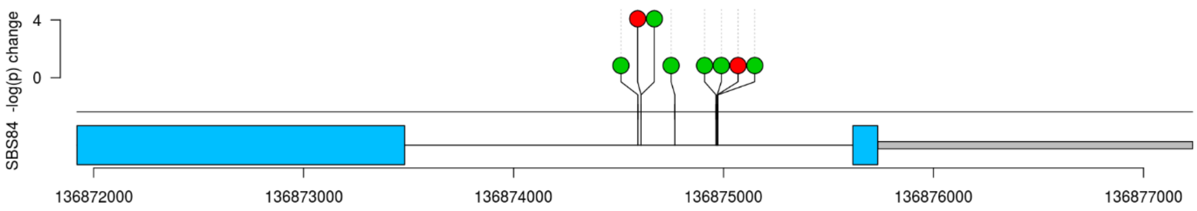

PIM1:

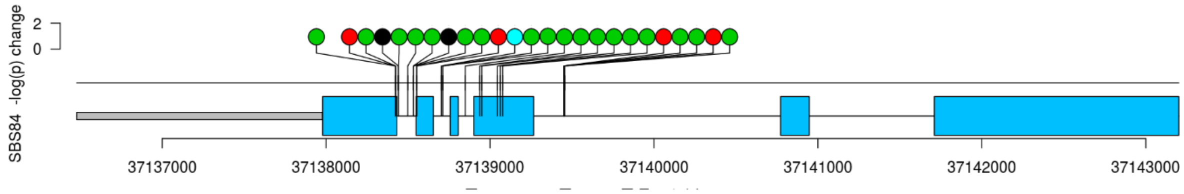

RHOH:

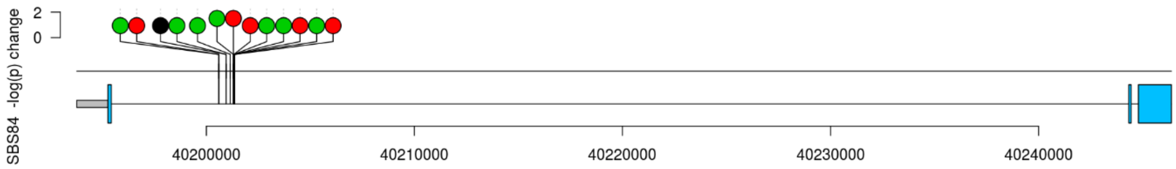

PAX5:

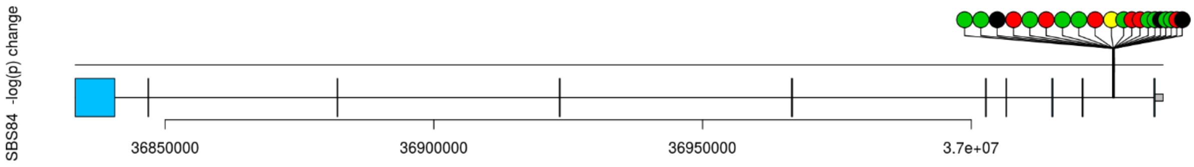

IGKV4-1:

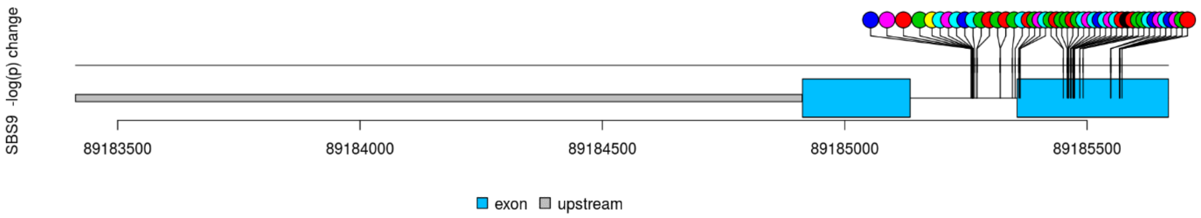
SBS9 -log(p) change
89183500
89184000
89184500
89185000
89185500
exon
upstream

Supplementary Figure 10. Translation table between NxSxN-extended signatures and COSMIC SBS signatures V3.

The table indicate mapping probabilities between NxSxN-extended signatures and COSMIC SBS signatures V3. Strength of probabilities were illustrated in red.

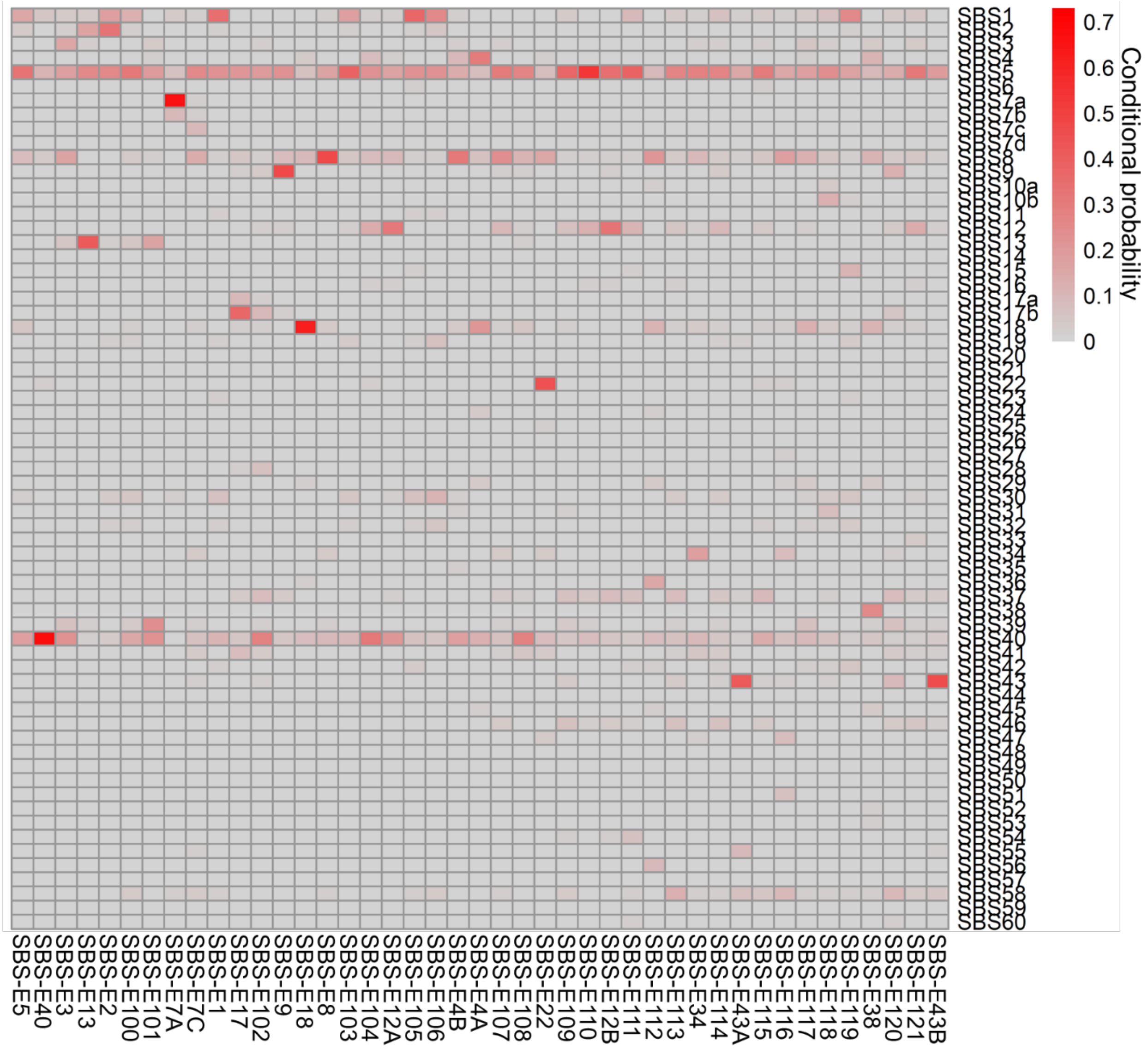

Supplementary Figure 11. Quantile-Quantile plot of association tests from various mutational signatures

(a) COSMIC SBS signatures V3

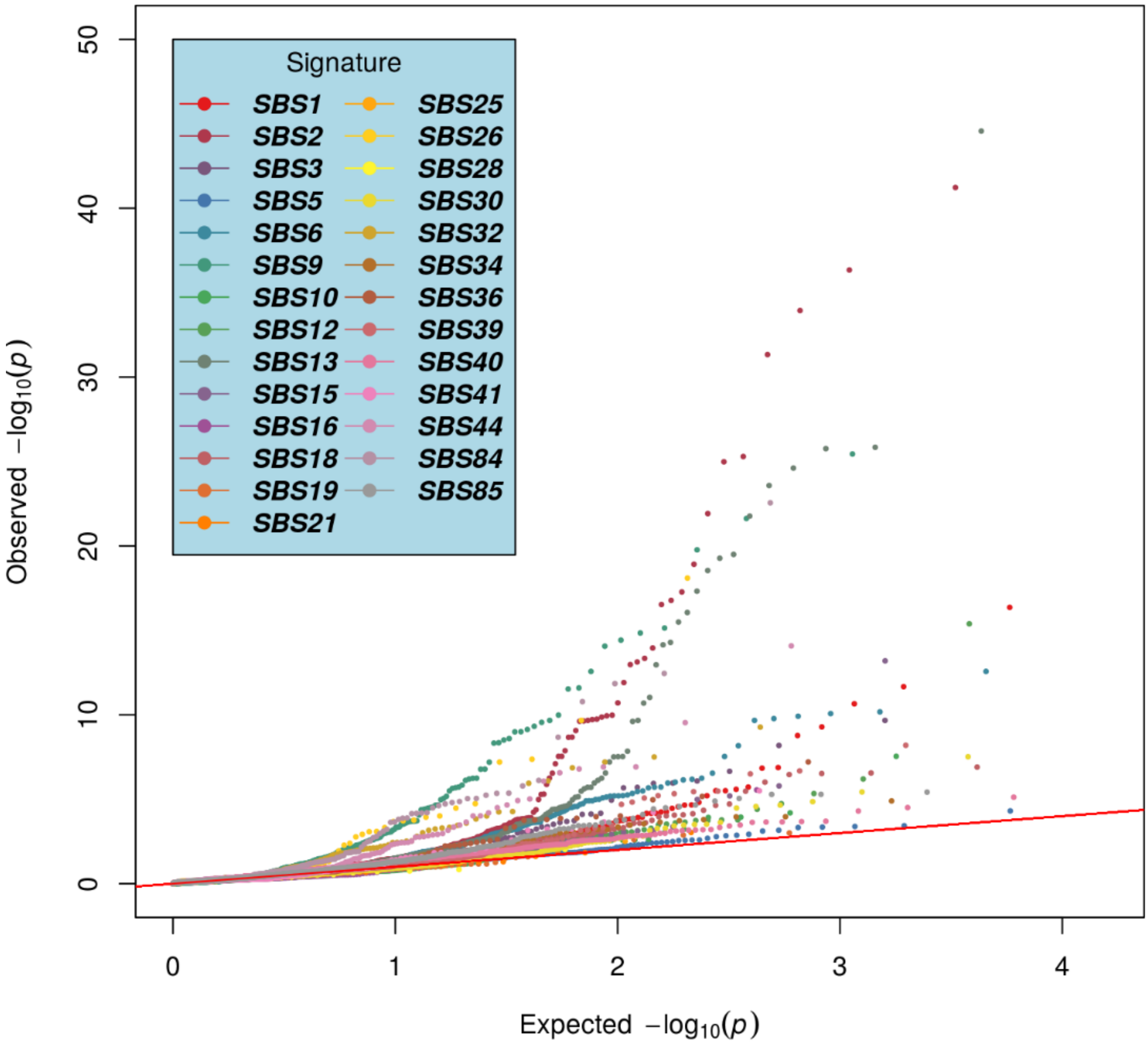

(b) NxSxN-extended signatures

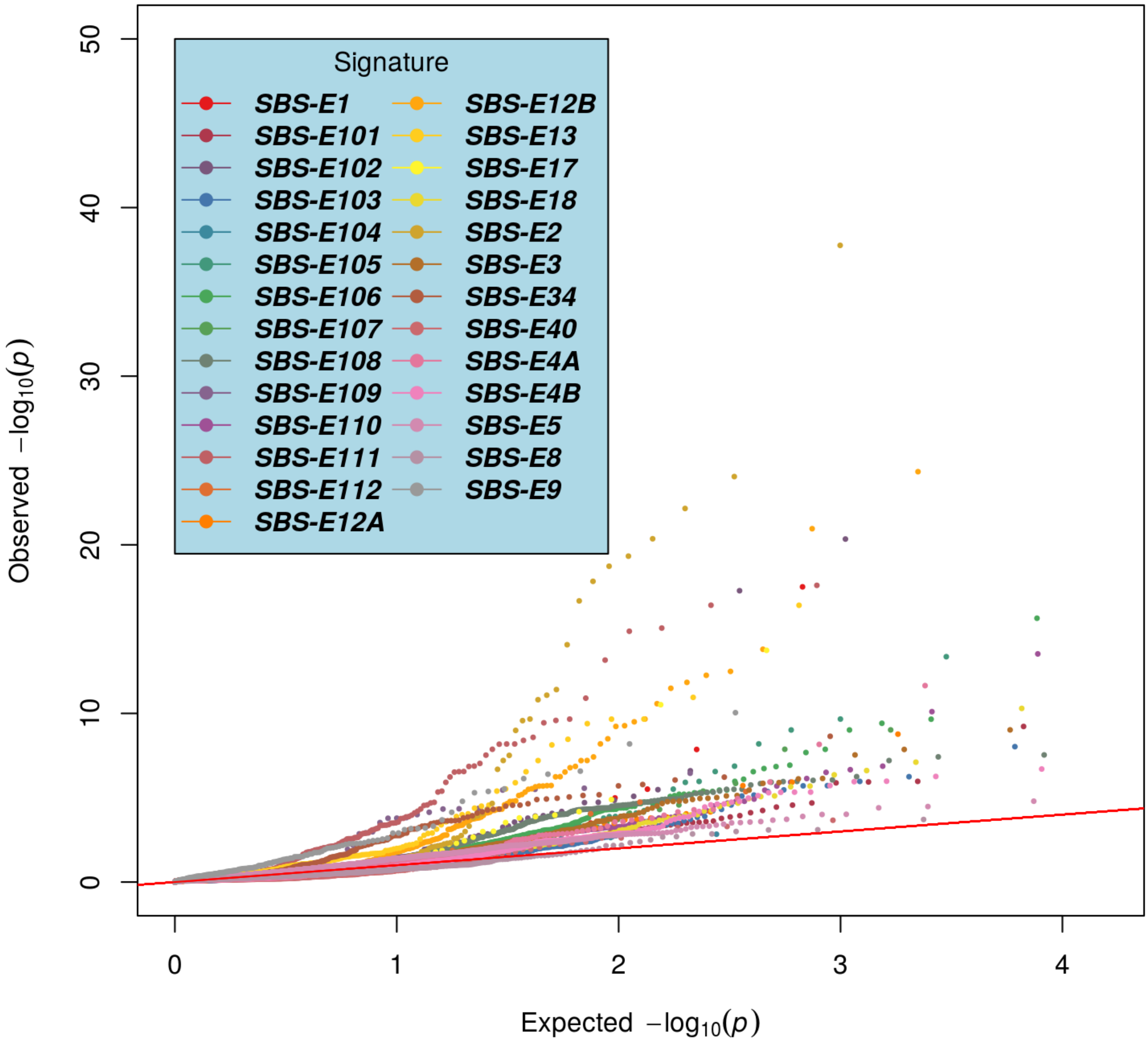

Supplementary Figure 12. Quantile-Quantile plot for differential gene expression tests using DESeq2

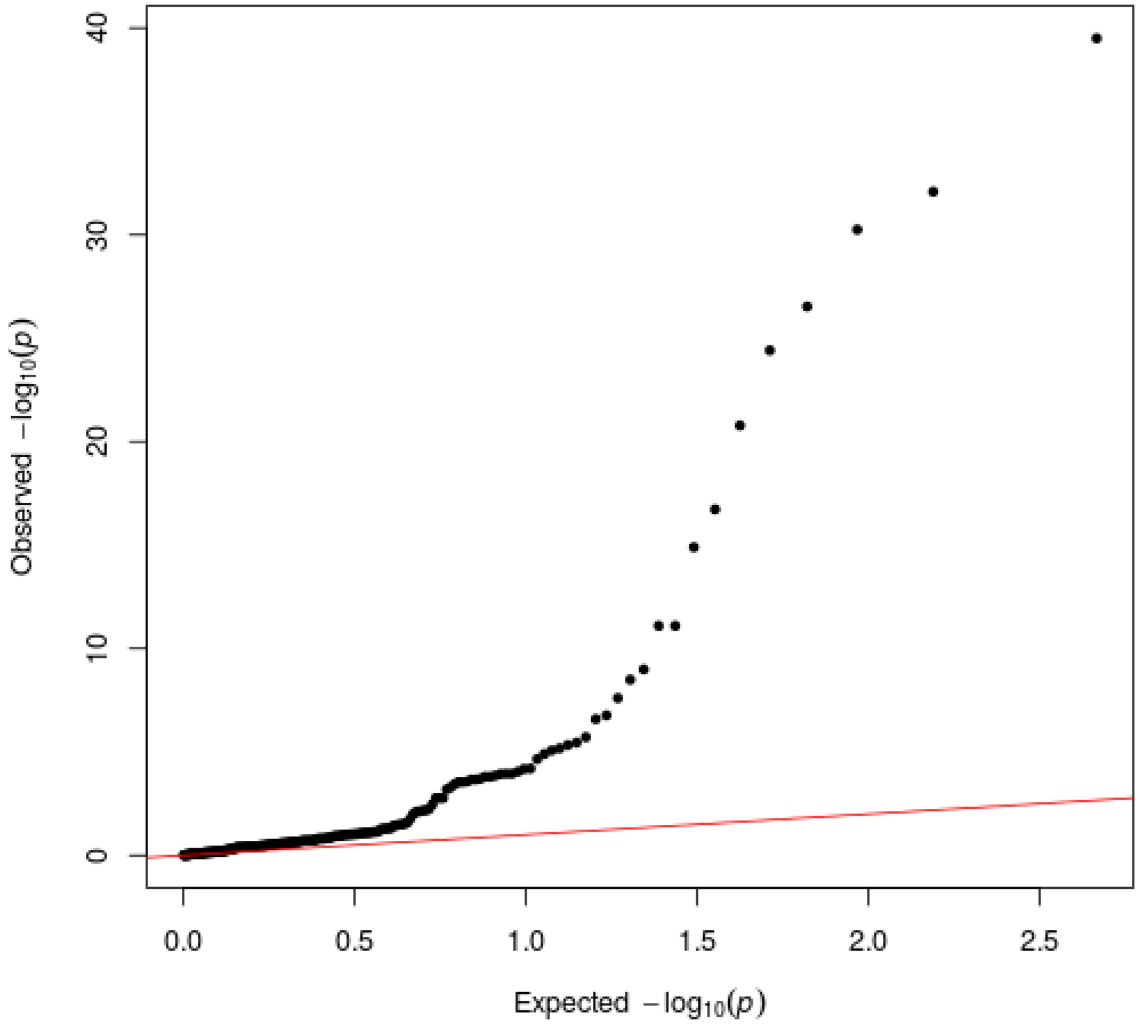

Supplementary Figure 13: Description of NxSxN-extended context and signatures

(a) Context information used by NxSxN-extended signatures in comparison with the cosmic SBS signatures

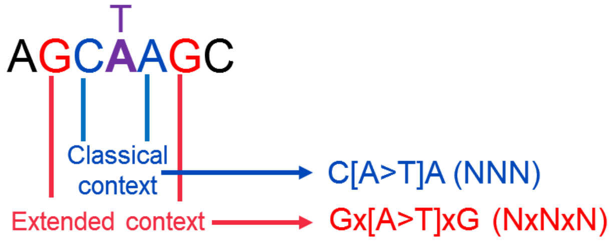

(b) The 96-class profiles of 43 NxSxN-extended signatures.

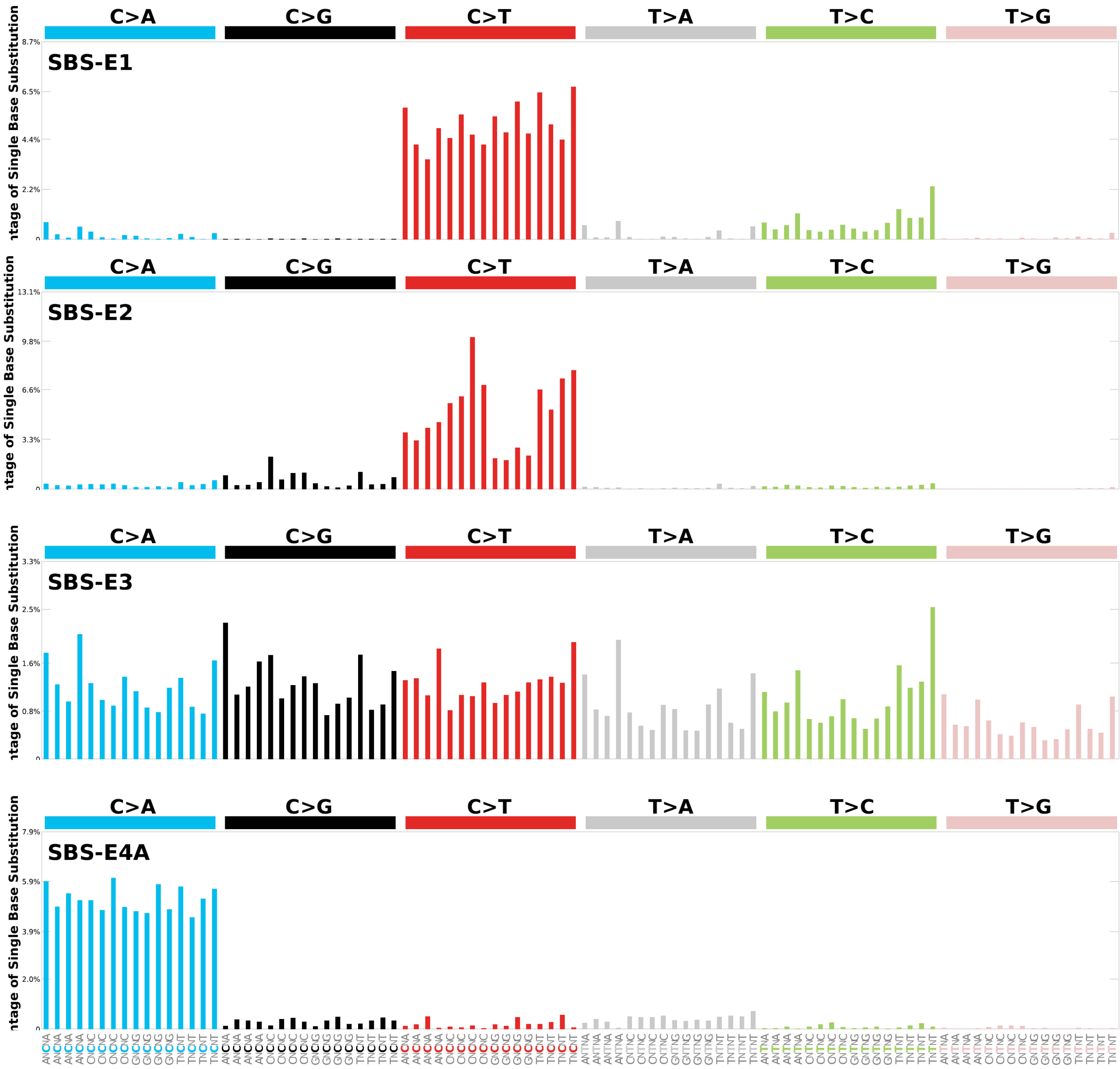

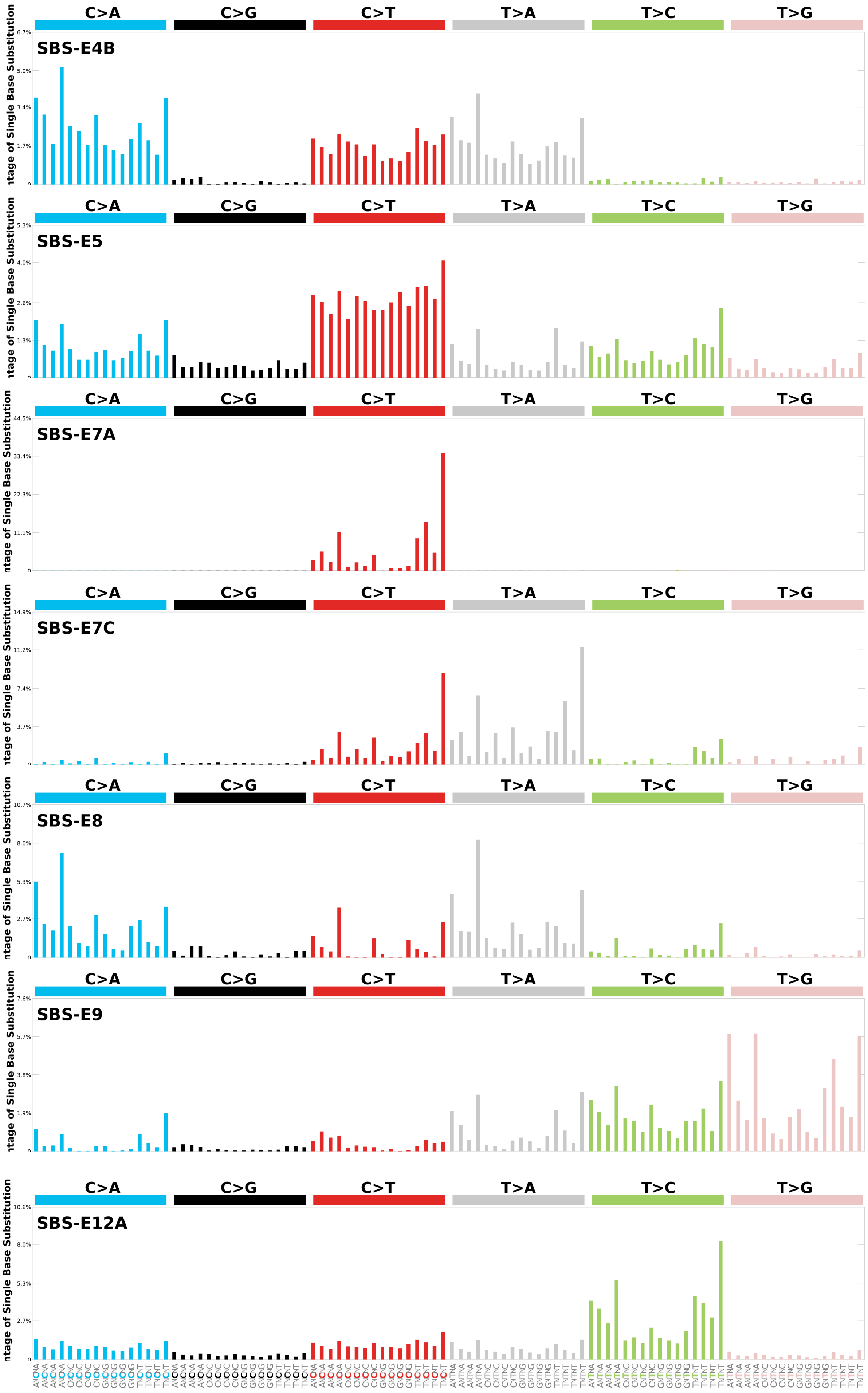

C>A
C>G
C>T
T>A
T>C
T>G
SBS-E4B
SBS-E5
SBS-E7A
SBS-E7C
SBS-E8
SBS-E9
SBS-E12A
ntage of Single Base Substitution
6.7%
5.0%
3.4%
1.7%
5.3%
4.0%
2.6%
1.3%
44.5%
33.4%
22.3%
11.1%
14.9%
11.2%
7.4%
3.7%
10.7%
8.0%
5.3%
2.7%
7.6%
5.7%
3.8%
1.9%
10.6%
8.0%
5.3%
2.7%
0

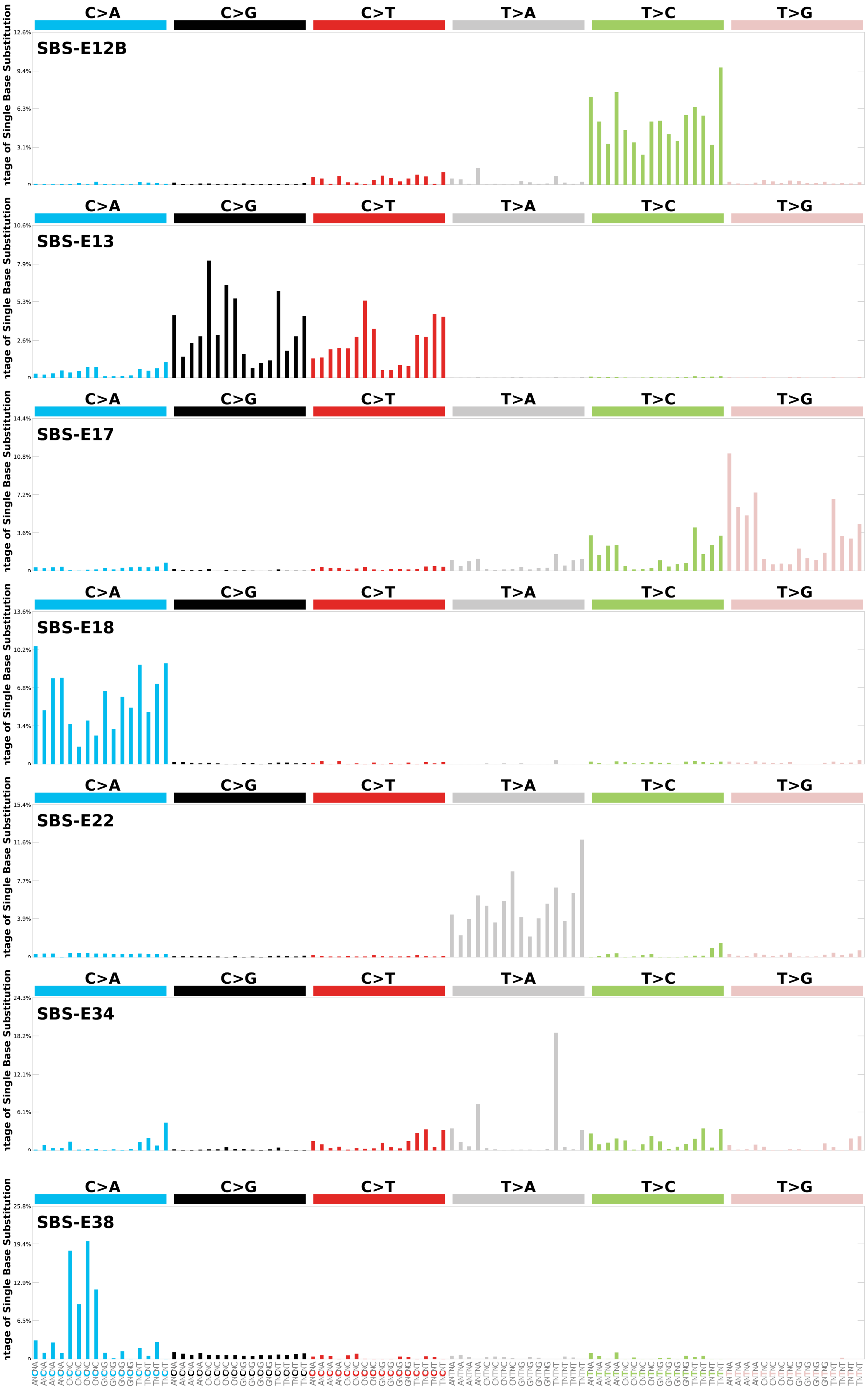
C>A
C>G
C>T
T>A
T>C
T>G
SBS-E12B
12.6%
9.4%
6.3%
3.1%
SBS-E13
10.6%
7.9%
5.3%
2.6%
SBS-E17
14.4%
10.8%
7.2%
3.6%
SBS-E18
13.6%
10.2%
6.8%
3.4%
SBS-E22
15.4%
11.6%
7.7%
3.9%
SBS-E34
24.3%
18.2%
12.1%
6.1%
SBS-E38
25.8%
19.4%
12.9%
6.5%
ntage of Single Base Substitution

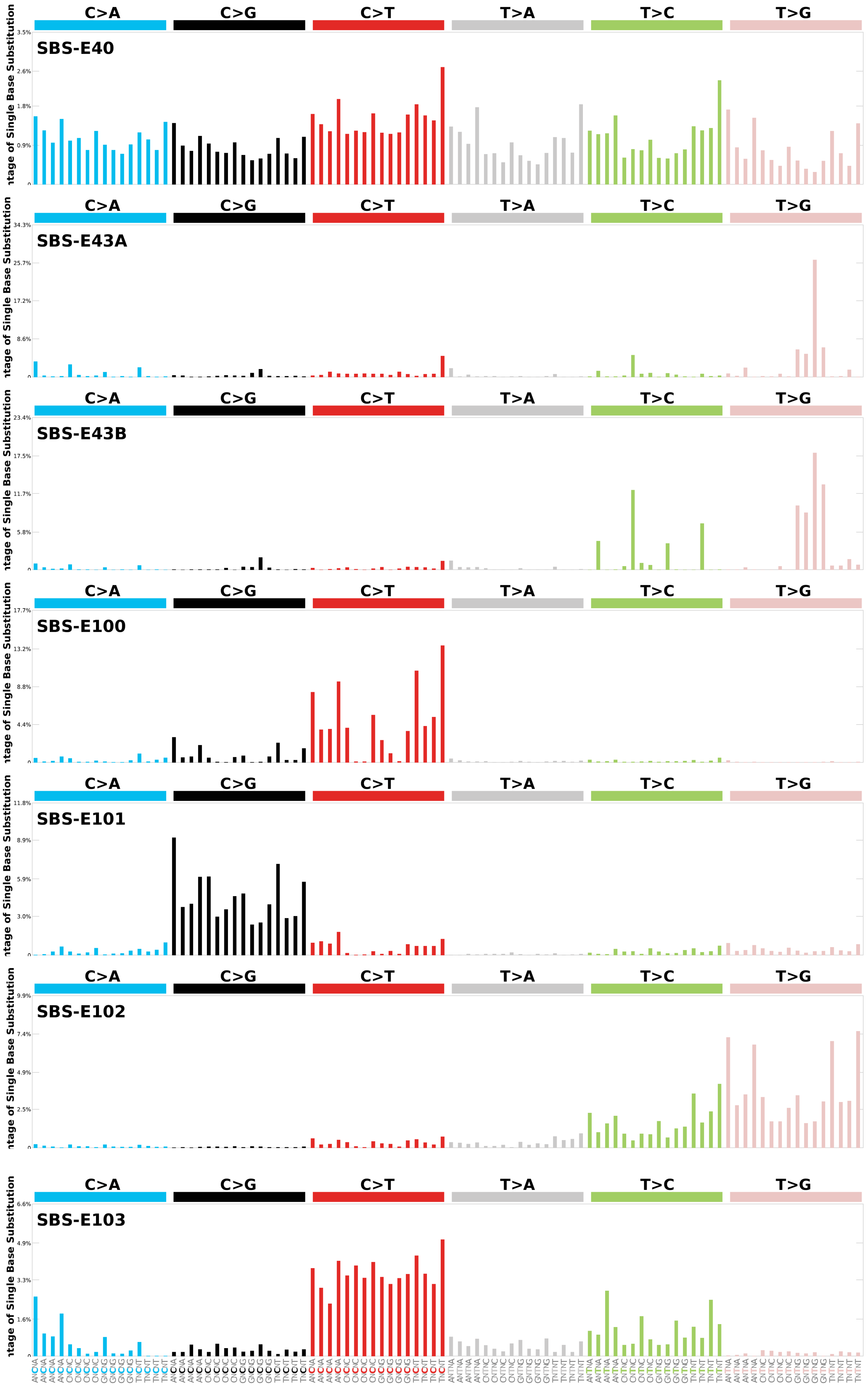
C>A
C>G
C>T
T>A
T>C
T>G
SBS-E40
3.5%
2.6%
1.8%
0.9%
0
SBS-E43A
34.3%
25.7%
17.2%
8.6%
0
SBS-E43B
23.4%
17.5%
11.7%
5.8%
0
SBS-E100
17.7%
13.2%
8.8%
4.4%
0
SBS-E101
11.8%
8.9%
5.9%
3.0%
0
SBS-E102
9.9%
7.4%
4.9%
2.5%
0
SBS-E103
6.6%
4.9%
3.3%
1.6%
0
ntage of Single Base Substitution

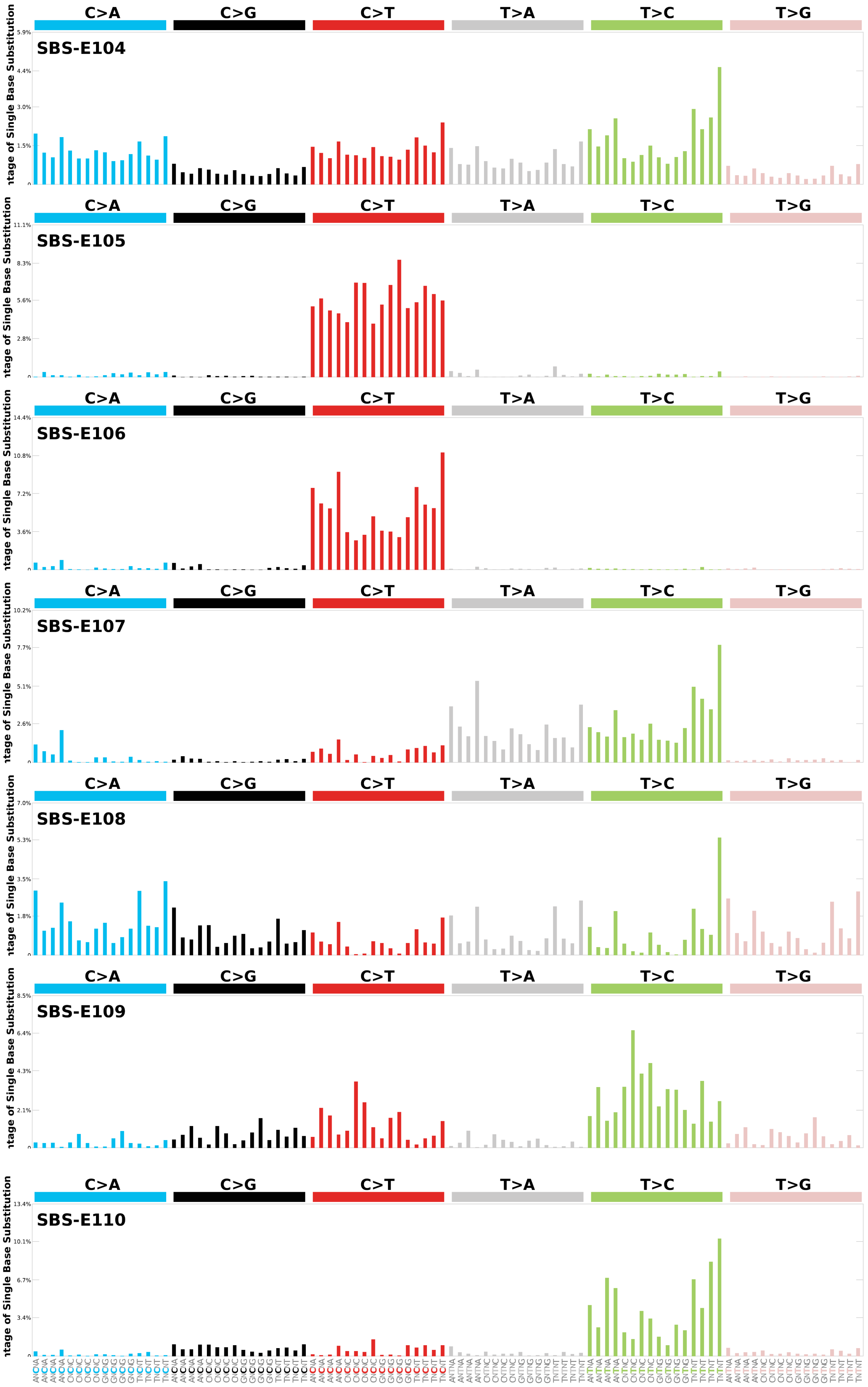
C>A
C>G
C>T
T>A
T>C
T>G
SBS-E104
5.9%
4.4%
3.0%
1.5%
0
SBS-E105
11.1%
8.3%
5.6%
2.8%
0
SBS-E106
14.4%
10.8%
7.2%
3.6%
0
SBS-E107
10.2%
7.7%
5.1%
2.6%
0
SBS-E108
7.0%
5.3%
3.5%
1.8%
0
SBS-E109
8.5%
6.4%
4.3%
2.1%
0
SBS-E110
13.4%
10.1%
6.7%
3.4%
0
ntage of Single Base Substitution

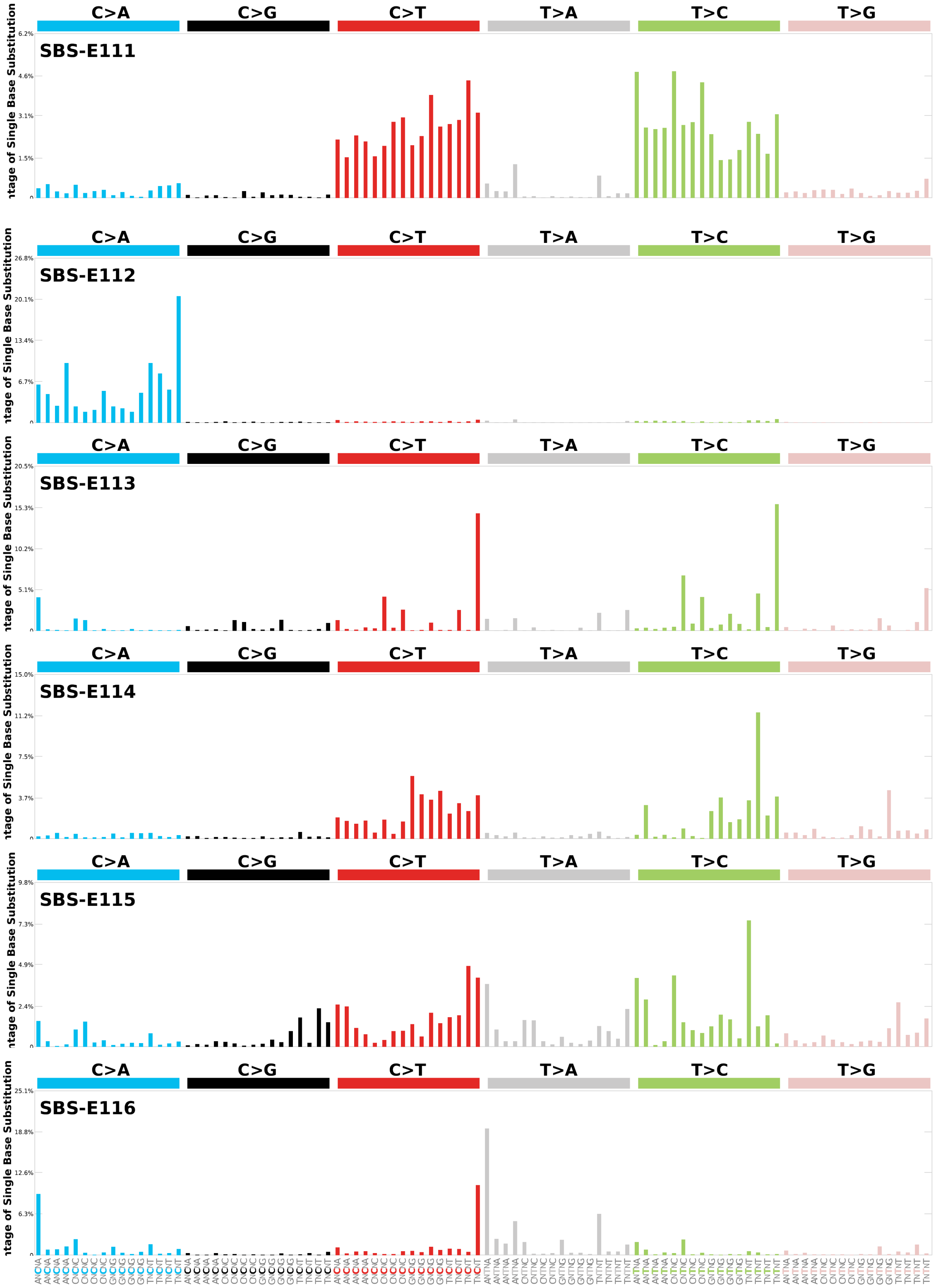

C>A
C>G
C>T
T>A
T>C
T>G
SBS-E111
6.2%
4.6%
3.1%
1.5%
0
SBS-E112
26.8%
20.1%
13.4%
6.7%
0
SBS-E113
20.5%
15.3%
10.2%
5.1%
0
SBS-E114
15.0%
11.2%
7.5%
3.7%
0
SBS-E115
9.8%
7.3%
4.9%
2.4%
0
SBS-E116
25.1%
18.8%
12.6%
6.3%
0

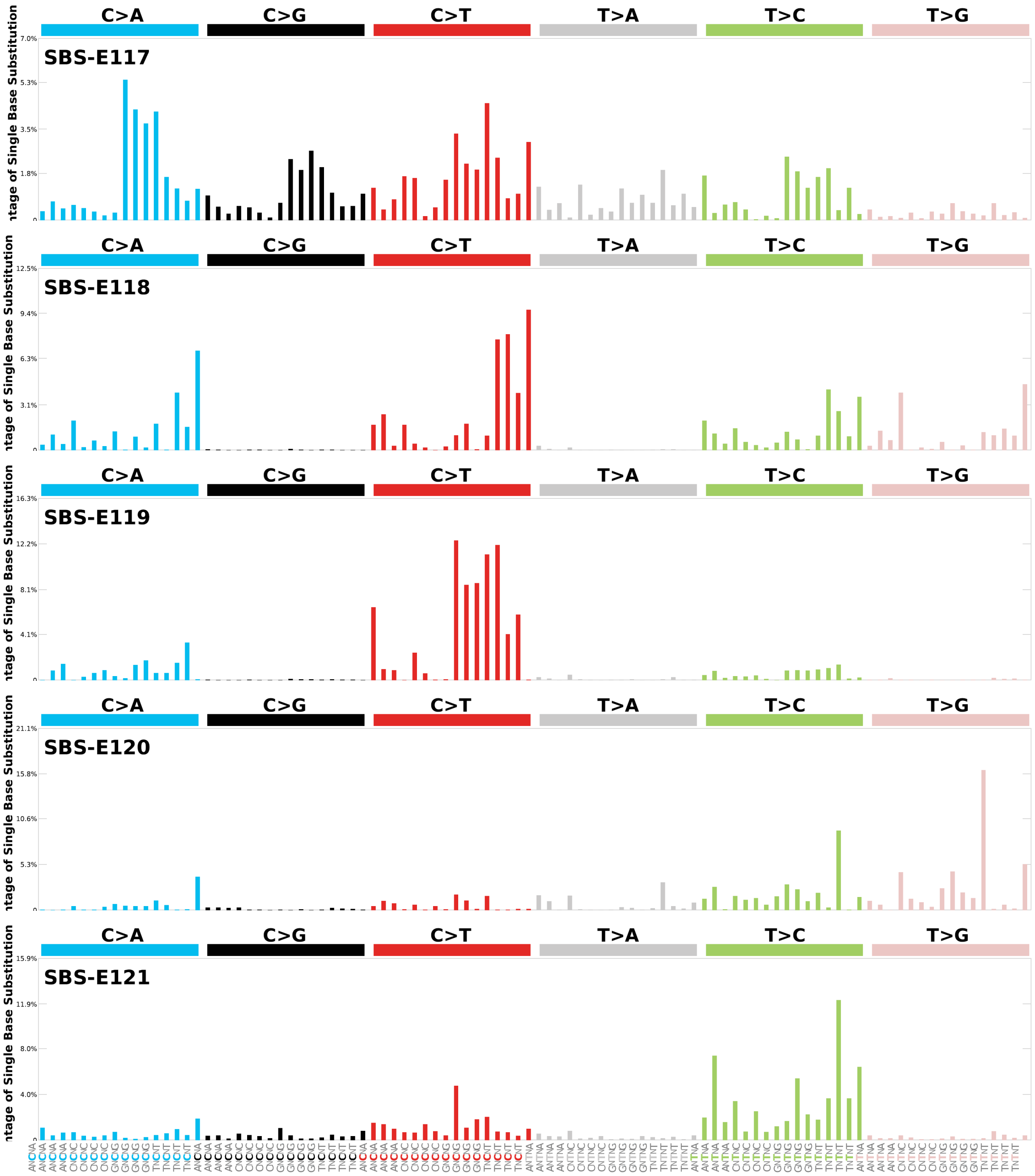

C>A
C>G
C>T
T>A
T>C
T>G
SBS-E117
SBS-E118
SBS-E119
SBS-E120
SBS-E121
ntage of Single Base Substitution

Supplementary Figure 14. Heatmap showing cosine similarity between signatures deciphered by signeR and sigProfiler

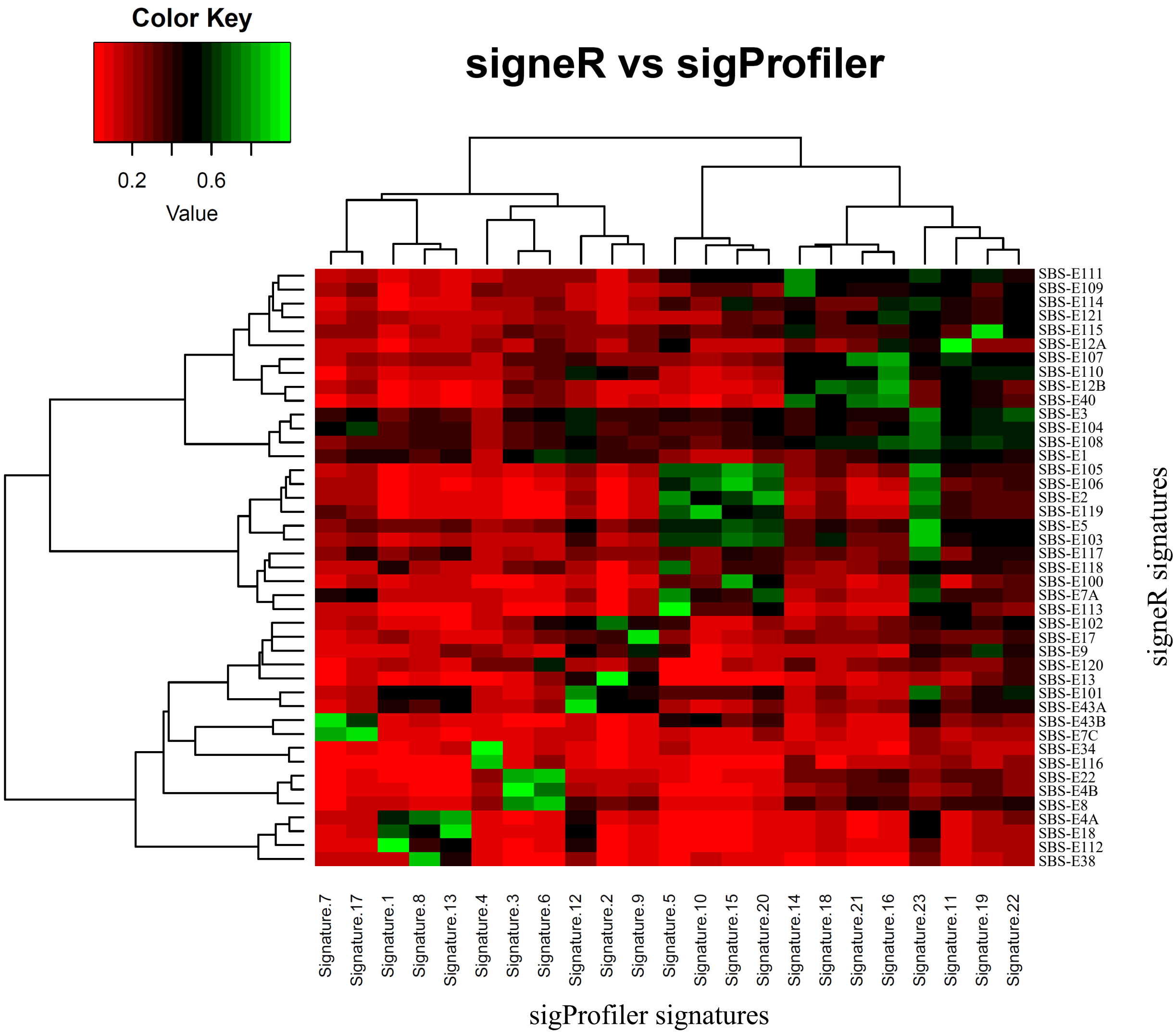

Supplementary Figure 15. Translation table between NxSxN-extended signatures and Pentanucleotide SBS signatures

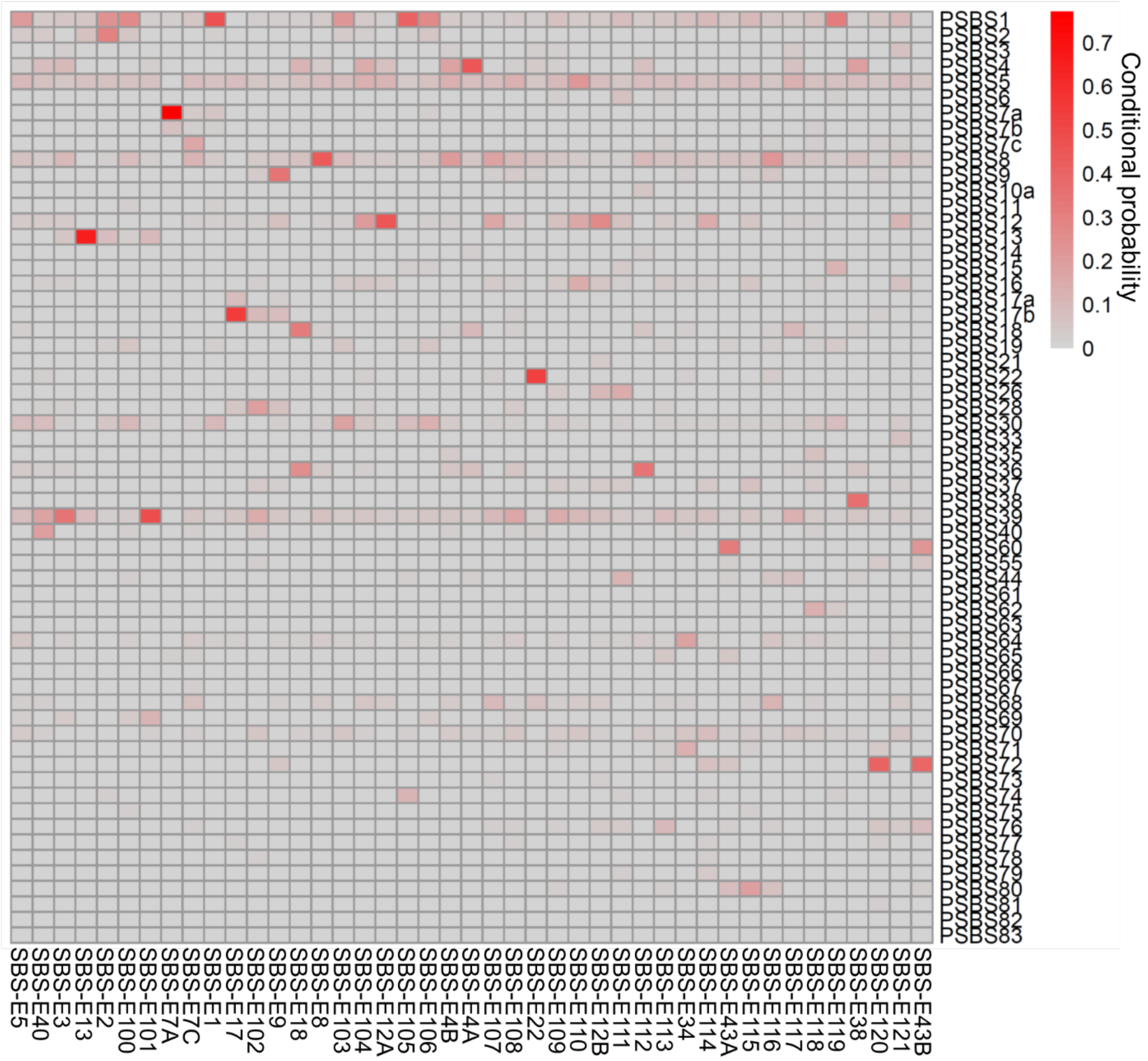

Supplementary Figure 16. The landscape of NxSxN-extended signature exposures across tumor entities.

The threshold for possible signature attribution of each sample is 5% where the proportion of tumor in each sample entity is depicted by the size of the circle. The median of normalized exposures indicated by the color of the circles.

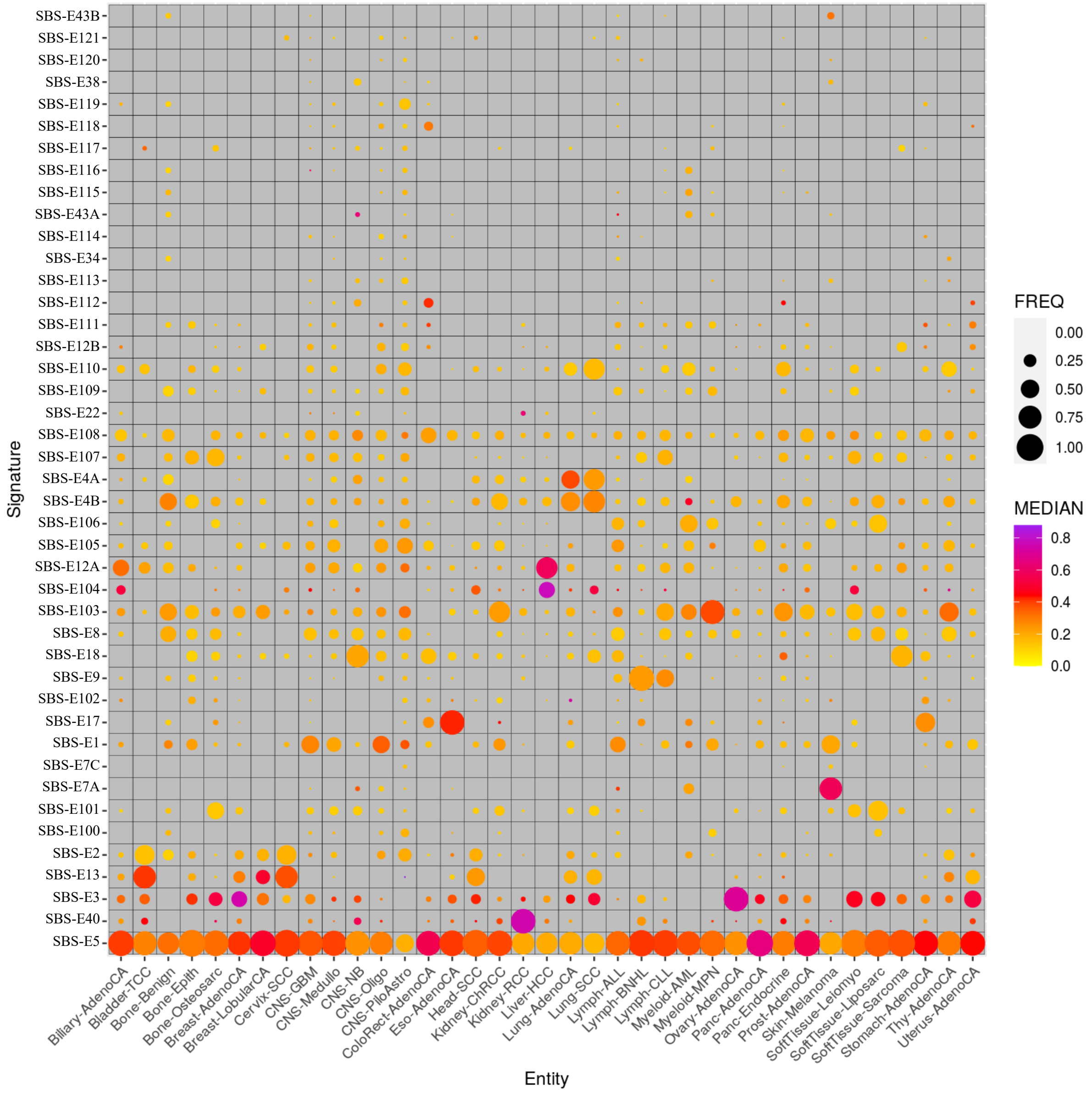

Supplementary Figure 17. The receiver operating characteristic curve (ROC) of describing the prediction performance of signature SBS-E3, signature SBS3 and the combined maximum.

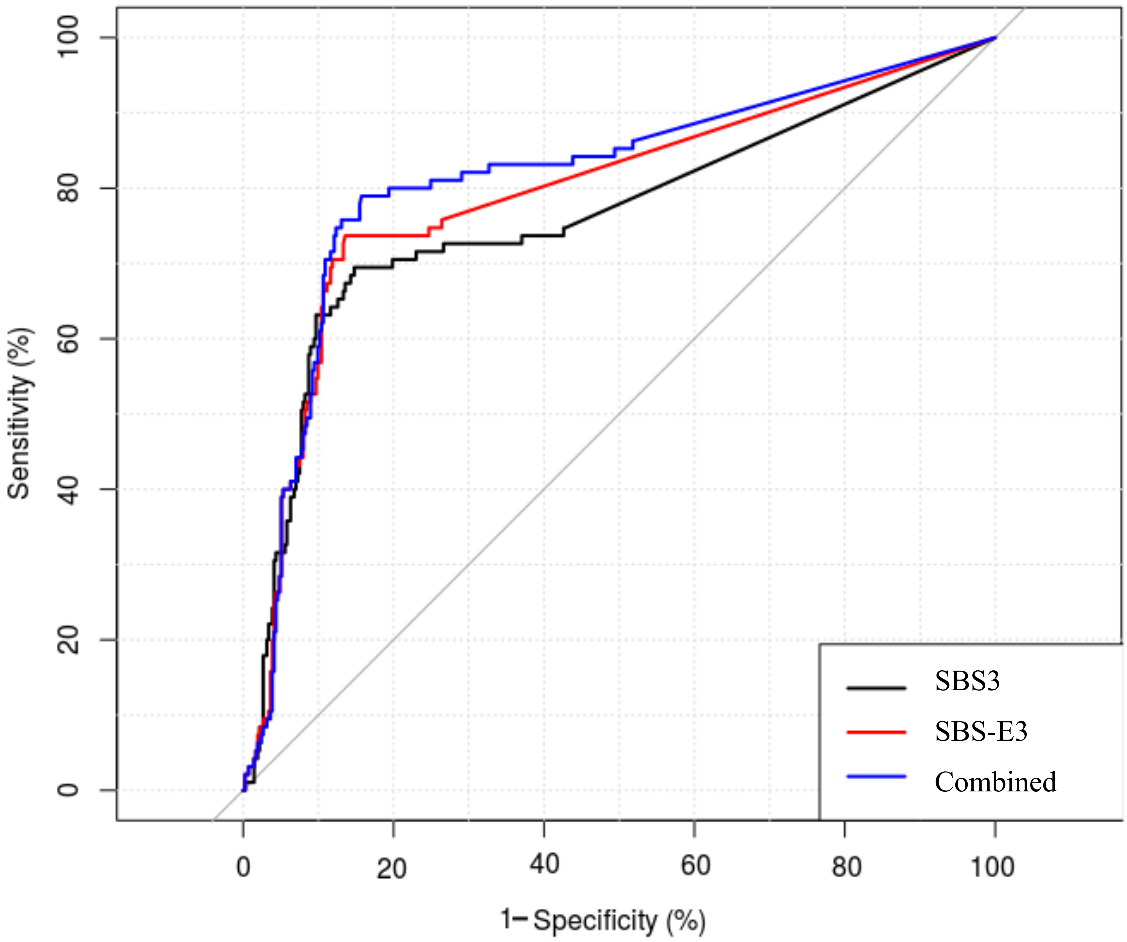

Supplementary Figure 18: The *IDH1*, *H3F3A* and *PTEN* coding hotspot mutations

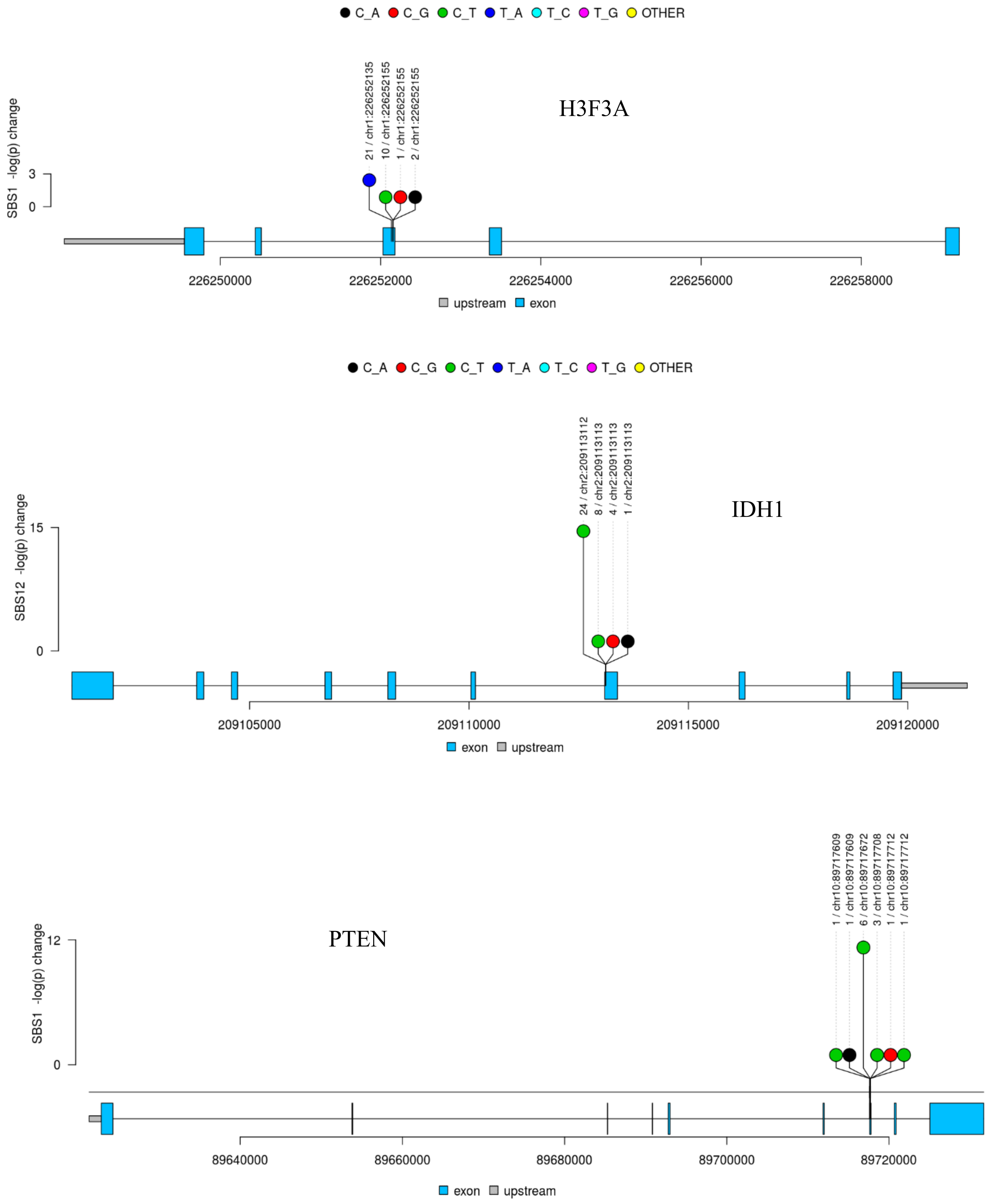

Supplementary Figure 19. Cosine similarity of simulated signature exposures and estimated exposures of SBS13 using penta-nucleotide signatures (PSBS) and NxSxN-extended signatures (SBS-E).

Based on six simulations using eight signatures present in trinucleotide and NxSxN-context (SBS2, SBS7a, SBS8, SBS12, SBS13, SBS17b, SBS22, SBS38). The boxes indicate the 25th and 75th percentiles with the median highlighted by a black line, whiskers extend to 1.5 times the interquartile range from the 25th and 75th percentiles.

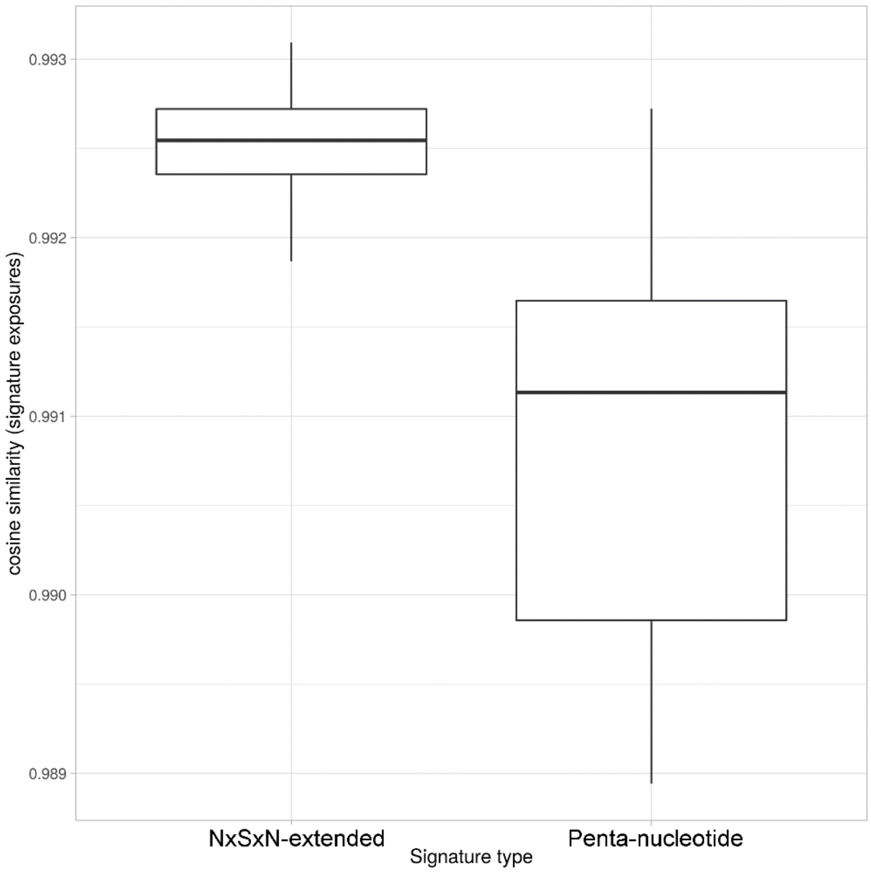

Supplementary Figure 20. *RAD51B* isoforms information and the hotspot

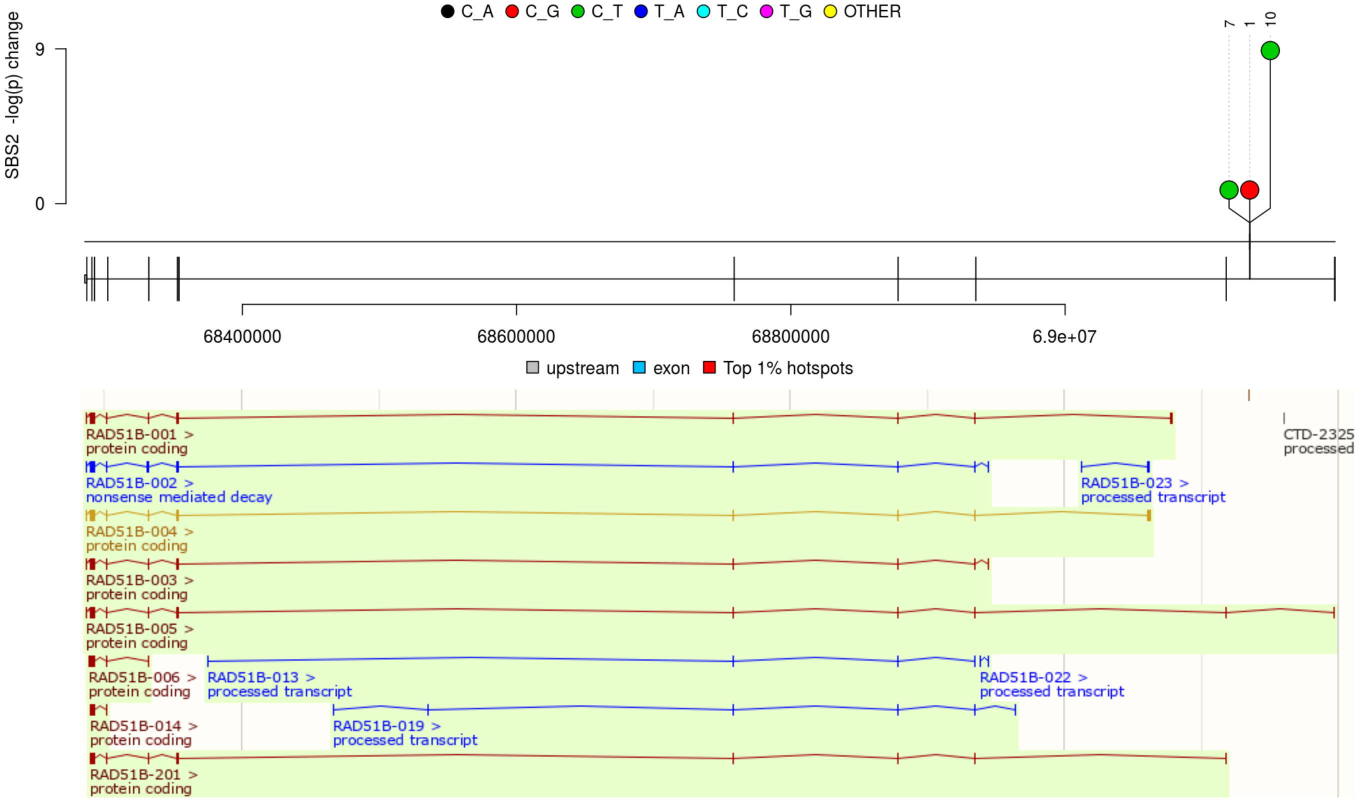

Supplementary Figure 21. Motif analysis of SBS17 hotspots

Rank	Motif	P-value	log P-pvalue	% of Targets	% of Background	STD(Bg STD)
1	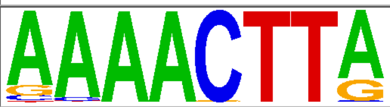	1e-136	-3.150e+02	37.81%	1.86%	1.0bp (2.5bp)
2	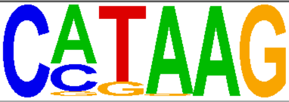	1e-52	-1.213e+02	17.53%	1.18%	1.0bp (2.7bp)
3	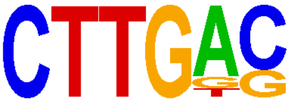	1e-19	-4.514e+01	11.51%	1.89%	2.0bp (3.1bp)
4 *	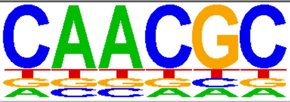	1e-1	-3.457e+00	0.27%	0.01%	0.0bp (1.0bp)

References

1 Alexandrov, L. B. *et al.* The repertoire of mutational signatures in human cancer. *Nature* **578**, 94-101, doi:10.1038/s41586-020-1943-3 (2020).

2 Davies, H. *et al.* HRDetect is a predictor of BRCA1 and BRCA2 deficiency based on mutational signatures. *Nat Med* **23**, 517-+, doi:10.1038/nm.4292 (2017).

3 Kim, J. *et al.* Somatic ERCC2 mutations are associated with a distinct genomic signature in urothelial tumors. *Nature genetics* **48**, 600-606, doi:10.1038/ng.3557 (2016).

4 Khodabakhshi, A. H. *et al.* Recurrent targets of aberrant somatic hypermutation in lymphoma. *Oncotarget* **3**, 1308-1319 (2012).

5 Rheinbay, E. *et al.* Analyses of non-coding somatic drivers in 2,658 cancer whole genomes. *Nature* **578**, 102-111, doi:10.1038/s41586-020-1965-x (2020).

6 Barutcu, A. R. *et al.* Chromatin interaction analysis reveals changes in small chromosome and telomere clustering between epithelial and breast cancer cells. *Genome biology* **16**, doi:10.1186/s13059-015-0768-0 (2015).
