# Supplementary material for: Association of mutation signature effectuating processes with mutation hotspots in driver genes and non-coding regions
Source: Nat Commun. 2022 Jan 10;13:178. doi: 10.1038/s41467-021-27792-6 (PMC8748499; doi:10.1038/s41467-021-27792-6)
Supplement: Supplementary file 4 — Reporting Summary [file 41467_2021_27792_MOESM4_ESM.pdf]

nature portfolio

Corresponding author(s): Marc Zapatka and John K.L. Wong

Last updated by author(s): 2021/11/15

Reporting Summary

Nature Portfolio wishes to improve the reproducibility of the work that we publish. This form provides structure for consistency and transparency in reporting. For further information on Nature Portfolio policies, see our Editorial Policies and the Editorial Policy Checklist.

Statistics

For all statistical analyses, confirm that the following items are present in the figure legend, table legend, main text, or Methods section.

n/a	Confirmed	
☐	☒	The exact sample size (*n*) for each experimental group/condition, given as a discrete number and unit of measurement
☐	☒	A statement on whether measurements were taken from distinct samples or whether the same sample was measured repeatedly
☐	☒	The statistical test(s) used AND whether they are one- or two-sided<br>*Only common tests should be described solely by name; describe more complex techniques in the Methods section.*
☐	☒	A description of all covariates tested
☐	☒	A description of any assumptions or corrections, such as tests of normality and adjustment for multiple comparisons
☐	☒	A full description of the statistical parameters including central tendency (e.g. means) or other basic estimates (e.g. regression coefficient) AND variation (e.g. standard deviation) or associated estimates of uncertainty (e.g. confidence intervals)
☐	☒	For null hypothesis testing, the test statistic (e.g. *F*, *t*, *r*) with confidence intervals, effect sizes, degrees of freedom and *P* value noted<br>*Give P values as exact values whenever suitable.*
☒	☐	For Bayesian analysis, information on the choice of priors and Markov chain Monte Carlo settings
☒	☐	For hierarchical and complex designs, identification of the appropriate level for tests and full reporting of outcomes
☐	☒	Estimates of effect sizes (e.g. Cohen's *d*, Pearson's *r*), indicating how they were calculated

Our web collection on statistics for biologists contains articles on many of the points above.

Software and code

Policy information about availability of computer code

Data collection	No software was used.
Data analysis	The analysis workflow involved signature fitting, signature exposures estimation, association tests and visualization. The individual components involved in the analysis are as follows: sigProfiler(v2.5.14), signeR(l.8.0), R(3.6.0), ggplot2(3.3.3), SKAT(2.0.1), VariantAnnotation (1.32.0), trackViewer(l.22.1), SignatureAnalyzer(1.1), ApoHP(initial1), ANNOVAR(2019Oct24), Deepbind(0.11), DESeq2(1.26.0), YAPSA(1.16.0), sigfit-NMF/sigfit-Emu(2.0.0) and umap (0.2.7.0). The driver discovery pipeline is available at https://github.com/wkljohn/sigDriver (DOI 10.5281/zenodo.5703087).

For manuscripts utilizing custom algorithms or software that are central to the research but not yet described in published literature, software must be made available to editors and reviewers. We strongly encourage code deposition in a community repository (e.g. GitHub). See the Nature Portfolio guidelines for submitting code & software for further information.

Data

Policy information about availability of data

All manuscripts must include a data availability statement. This statement should provide the following information, where applicable:

- Accession codes, unique identifiers, or web links for publicly available datasets
- A description of any restrictions on data availability
- For clinical datasets or third party data, please ensure that the statement adheres to our policy

Somatic variant calls from PCAWG and ICGC were obtained from the ICGC web portal (https://dcc.icgc.org/api/v1/download?fn=/PCAWG/consensus_snv_indel/final_consensus_passonly.snv_mnv_indel.icgc.public.maf.gz, s3://pcawg-tcga/consensus_snv_indel/final_consensus_passonly.snv_mnv_indel.tcga.controlled.maf.gz), the TCGA section of the dataset is under controlled access (https://dcc.icgc.org/releases/PCAWG/

consensus_snv_indel) , access can be requested through https://dbgap.ncbi.nlm.nih.gov/. Download details are provided at http://docs.icgc.org/pcawg/data/#download-from-pdc . Somatic variant calls from the pediatric cancer cohort were obtained from the R2 database (https://hgserver1.amc.nl/cgi-bin/r2/main.cgi?&dscope=DKFZ_PED&option=about_dscope). Tumors from the CATCH cohort were collected by National Center for Tumor Diseases (NCT) in Heidelberg and were processed by ODCF using DKFZ whole genome sequencing and transcriptome sequencing pipelines. Sequencing data for CATCH can be found at EGA under accession ID: EGAD00001007563 [https://ega-archive.org/datasets/EGAD00001007563]. The CATCH dataset is under controlled access, please contact hipo_daco@dkfz-heidelberg.de to request for access permission. Data sets described specifically in this manuscript can be found in the supplementary data. Source data are provided with this paper.

Field-specific reporting

Please select the one below that is the best fit for your research. If you are not sure, read the appropriate sections before making your selection.

☒ Life sciences ☐ Behavioural & social sciences ☐ Ecological, evolutionary & environmental sciences

For a reference copy of the document with all sections, see nature.com/documents/nr-reporting-summary-flat.pdf

Life sciences study design

All studies must disclose on these points even when the disclosure is negative.

Sample size	No sample size calculation was performed. We included the variant calls from three large consistently called cohorts from ICGC, PCAWG and pedpancan resulting in 3813 tumors for driver discovery and 5070 tumors for signature discovery. Furthermore, data from 258 breast cancer tumors originating from metastatic breast cancer patients (CATCH) were used for replication.
Data exclusions	We have excluded 121 repeated measurements of the same patients from the PCAWG dataset.
Replication	In order to evaluate the reproducibility of our approach, we performed analysis on an independent dataset of 258 metastatic breast cancers. The cohort replicated 5 of the 6 hotspots from APOBEC signatures.
Randomization	No randomisation was performed as we included all possible samples meeting the criteria explained above.
Blinding	Investigators were not blinded as it is typically not used in the field. For the study identifying driver gene candidates in an exploratory way blinding is not necessary because the results are quantitative and did not require subjective judgment or interpretation.

Reporting for specific materials, systems and methods

We require information from authors about some types of materials, experimental systems and methods used in many studies. Here, indicate whether each material, system or method listed is relevant to your study. If you are not sure if a list item applies to your research, read the appropriate section before selecting a response.

Materials & experimental systems

n/a	Involved in the study	
☒	☐	Antibodies
☒	☐	Eukaryotic cell lines
☒	☐	Palaeontology and archaeology
☒	☐	Animals and other organisms
☐	☒	Human research participants
☒	☐	Clinical data
☒	☐	Dual use research of concern

Methods

n/a	Involved in the study	
☒	☐	ChIP-seq
☒	☐	Flow cytometry
☒	☐	MRI-based neuroimaging

Human research participants

Policy information about studies involving human research participants

Population characteristics	For the population characteristic of the PCAWG samples, refer to the PCAWG publication. The population characteristic of the CATCH cohort and the pediatric cancer cohort are not available.
Recruitment	Patients were recruited by the participating centres following local protocols. As different numbers of patients from the individual cancer entities are included in the data set this distribution introduces a bias that we controlled by performing also cancer entity specific analyses.
Ethics oversight	For the PCAWG part of the data refer to TCGA Program Office and the Ethics and Governance Committee of the ICGC. Each individual ICGC and TCGA project that contributed data to PCAWG had their own local arrangements for ethics oversight and regulatory alignment. For the CATCH cohort refer to EGA accession ID EGAS00001004662.

Note that full information on the approval of the study protocol must also be provided in the manuscript.
